# A reversible and robust hybrid image steganography framework using radon transform and integer lifting wavelet transform

**DOI:** 10.1038/s41598-025-98539-2

**Published:** 2025-05-05

**Authors:** B. M. El-den, Walid Raslan

**Affiliations:** Electronics and Communications Engineering Department, Faculty of Engineering, Delta University for Science and Technology, Gamasa, 11152 Egypt

**Keywords:** Digital Steganography, Concealment of Data, Data Retrievability, Radon Transformation ($\text{RT})$, Integer Lifting Wavelet transformation ($\text{ILWT}$), Arithmetic Coding, Robustness, Electrical and electronic engineering, Computer science

## Abstract

This study introduces a reliable and reversible image steganography framework that integrates Radon Transform (RT) and Integer Lifting Wavelet Transform (ILWT) to enhance security, imperceptibility, and robustness. The RT obscures data locations through rotations, scaling, and translations, while ILWT ensures full reversibility by maintaining integer wavelet coefficients, guaranteeing no data loss during reconstruction. Data embedding is performed in the middle bit planes of high-frequency sub-bands (LH, HL, HH) using Arithmetic Coding, optimizing space utilization while preserving visual quality. Experimental results demonstrate the method’s superior performance, achieving Peak Signal-to-Noise Ratio (PSNR) values exceeding 46 dB, a 15% improvement over existing techniques, ensuring the hidden data remains imperceptible to the human eye. The system exhibits exceptional robustness, with a Bit Error Rate (BER) as low as 0.0017 under scaling distortions, representing a 30% reduction compared to state-of-the-art methods. Additionally, the framework achieves a 25% increase in embedding capacity, enabling higher data payloads without compromising image quality. High Normalized Correlation Coefficient (NCC) values close to 1 further confirm the algorithm’s reversibility and integrity, even under common attacks such as Gaussian Noise, Poisson Noise, Blurring, and Cropping. Compared to existing techniques, the proposed framework demonstrates significant improvements in embedding capacity, robustness, and visual fidelity. This method holds great promise for secure data hiding in applications such as secure communication, medical imaging, and digital watermarking, where data integrity and confidentiality are critical.

## Introduction

Secure data transmission over digital networks has become increasingly challenging due to the widespread digitization of information and communication networks. As a result, robust techniques are required to ensure both security and imperceptibility in data exchange. Steganography, the practice of concealing data within a cover medium such as an image, has emerged as a key solution for secure data transmission. Among steganographic techniques, reversible data hiding is particularly promising, as it allows both the original cover image and the hidden data to be fully recovered without loss. However, existing steganographic techniques face significant challenges in balancing imperceptibility, robustness, and reversibility—three essential factors that define the effectiveness of a steganographic system.

Imperceptibility ensures that the embedded data remains visually indistinguishable from the original cover image, typically measured by metrics such as Peak Signal-to-Noise Ratio (PSNR) and Structural Similarity Index Measure (SSIM). Robustness refers to the system’s ability to resist image processing attacks such as noise addition, compression, and geometric transformations. Reversibility guarantees that the cover image can be perfectly restored after data extraction, which is particularly crucial in applications such as medical imaging, secure communications, and digital watermarking.

Traditional steganographic techniques such as Least Significant Bit (LSB) embedding are widely used due to their simplicity and high embedding capacity. However, LSB-based methods are highly vulnerable to attacks, including geometric transformations and noise addition, making them unsuitable for high-security applications. As a result, frequency-domain techniques, such as Discrete Cosine Transform (DCT) and Discrete Wavelet Transform (DWT), have been explored to improve robustness by embedding data in transform coefficients rather than direct pixel values. While DWT-based methods have shown strong resilience against image-processing attacks, their reliance on floating-point coefficients introduces precision loss during reconstruction, which compromises reversibility. This limitation highlights the need for alternative approaches that can ensure both robustness and reversibility. To address this, Integer Wavelet Transform (IWT) has been proposed as an alternative since it operates with integer coefficients, ensuring lossless recovery of the cover image. Additionally, Radon Transform (RT) has gained attention for its ability to resist rotation, scaling, and translation (RST) attacks, making it highly suitable for robust steganographic applications. The integration of IWT and RT addresses the shortcomings of traditional methods by combining reversibility with enhanced resistance to geometric distortions. However, existing hybrid approaches combining RT and wavelet transforms often fail to achieve a balance between embedding capacity, imperceptibility, and robustness. This study addresses these limitations by proposing a novel hybrid framework that integrates RT and ILWT), offering enhanced robustness, reversibility, and embedding capacity.

In recent years, the field of steganography has witnessed significant advancements, driven by the growing demand for secure data transmission and robust, reversible data hiding techniques. Traditional methods, such as Least Significant Bit (LSB) embedding in the spatial domain, remain popular due to their simplicity and high embedding capacity. However, these methods are highly vulnerable to image-processing attacks, including noise addition, compression, and geometric transformations, which limit their applicability in secure communication and medical imaging^1–11^.

Recent advancements in steganography have focused on addressing key challenges such as embedding capacity, robustness, and imperceptibility. These advancements can be broadly categorized into two themes: methods that prioritize embedding capacity and those that emphasize robustness against attacks. In the context of embedding capacity, Sahu and Swain (2019) proposed a multi-stego-image based data hiding method that generates four stego-images from a single original image, significantly increasing embedding capacity while maintaining low distortion. Their method employs a modified LSB matching technique, achieving an average PSNR of over 36 dB across the stego-images. This approach demonstrates that multi-stego-image strategies can effectively balance embedding capacity and imperceptibility, addressing one of the key limitations of traditional LSB-based methods^12^. On the other hand, advancements in robustness have focused on resisting common image-processing attacks. For instance, Sahu and Swain (2019) introduced a steganography method based on Pixel Value Differencing (PVD) and Modulus Function (MF), which improves PSNR and embedding capacity while avoiding the fall-off boundary problem (FOBP). Their method demonstrates robustness against RS attacks and provides a good balance between imperceptibility and capacity^13^. Similarly, Sahu et al. (2021) proposed a multi-directional PVD and MF-based steganography technique that addresses both FOBP and incorrect extraction problem (IEP). By exploiting horizontal, vertical, and diagonal pixel differences, their method achieves high embedding capacity and robustness against RS attacks, salt & pepper noise, and PDH analysis^14^.

When confidential information is to be inserted within a cover image, the two topmost considerations are: one, that a generated embedded image must be of acceptable quality so that distortions caused by data embedding remain imperceptible, and two, that this embedded image should ensure the robustness of the embedded image. In other words, the data embedded into it must allow recovery, even after attacks during transmissions. Using all the above aspects, one has to say that embedding data into the frequency domain is much popular than spatial domain techniques for all the above reasons. These techniques manipulate the cover image by altering its frequency coefficients using a specific transformation function. Frequency domain methods are both imperceptible and resilient against geometric transformations and external attacks^15^.

Frequency domain methods derive the transformation coefficients of the original cover image using techniques such as the integer cosine transform^16^ or the Integer Wavelet Transform (IWT)^17^. Subsequently, these coefficients are adjusted to embed the secret data bit by bit. For instance, a reversible data hiding method based on Discrete Cosine Transformation (DCT) conceals a secret bit between adjacent DCT coefficients within the same image block^18^. Similarly, the Discrete Wavelet Transform (DWT) embeds the secret payload in the high-frequency coefficients by exploiting the statistical characteristics of the cover image. Although DWT demonstrates strong resilience against various image processing attacks, its reliance on floating-point coefficients may result in data loss during the reconstruction of the cover image. This limitation is mitigated by employing the IWT.

Transformation methods like the integer cosine transform^16^ or the IWT^17^ are used in frequency domain techniques to calculate the transform coefficients of the original cover image. These values are in turn modified with secret data. For instance, the $DCT$ hiding of a secret bit is that one secret bit in two neighboring $DCT$ coefficients within an image block is hidden^18^. Another such technique is where a discrete transformation (here, a $DWT$ technique) can hide the secret payload inside high-frequency coefficients, exploiting the statistical characteristics of the cover image^19^. $DWT$ has strong survivability under many attacks concerning image processing, but it provides floating-point coefficients, which may cause loss of data during reconstruction of the cover image. This is resolved through the $IWT$.

In $IWT$, secret data is embedded in the middle frequency through histogram modification, whereby it is processed in the wavelet domain using the cover image^17^. Moreover, embedding has been done in the Radon domain, which has the most substantial improvement regarding the bit error rate. A Radon-based method has been used to allow the concealed data translation invariance properties^20^. RST invariant watermarking employs Fourier transforms, converting them into log-polar coordinates, making them resistant to rotation, scaling, and translation attacks^21^.

Recent studies have explored the integration of hybrid transforms to combine the strengths of multiple techniques. For example, Amsaveni et al. (2024) proposed a hybrid steganography framework using RT and Discrete Fourier Transform (DFT), achieving robustness against geometric transformations. The RT’s ability to handle rotations and scaling by projecting images into a domain resistant to such attacks has made it a promising tool for robust steganography. However, these methods often lack reversibility, a critical requirement for applications such as medical imaging and secure communication, where data integrity is paramount^7^.

Despite these advancements, several challenges persist in existing steganographic frameworks. First, many frequency-domain techniques, such as DWT, produce floating-point coefficients, leading to data loss during reconstruction^6^. In addition to embedding capacity and imperceptibility, robustness against attacks is a critical factor in steganographic systems. Recent studies, such as the work by Sahu 2022 on digital watermarking techniques for multimedia authentication, have provided a comprehensive analysis of various watermarking methods, emphasizing their effectiveness in ensuring data integrity and authentication. Their findings highlight the importance of combining multiple techniques, such as frequency-domain transformations and hybrid approaches, to achieve robust and secure data hiding. These insights are particularly relevant for applications requiring high levels of security, such as medical imaging and secure communication^22^.

Second, while hybrid approaches combining RT and wavelet transforms have been proposed, they often fail to achieve a balance between embedding capacity, imperceptibility, and robustness. For instance, Abadi & Moallem (2022) proposed a robust watermarking method using RT and Contourlet Transform, but the method suffered from high computational complexity and limited embedding capacity. Third, most existing techniques do not adequately address the trade-off between robustness against geometric attacks and reversibility, limiting their applicability in real-world scenarios^3,23^.

To address these gaps, this study proposes a hybrid steganography framework that combines RT and ILWT. The RT ensures robustness against geometric transformations, while the ILWT guarantees reversibility by maintaining integer coefficients throughout the transformation process. By embedding data in the middle bit planes of high-frequency sub-bands (LH, HL, HH) using Arithmetic Coding, the proposed method optimizes embedding capacity without compromising visual quality. Furthermore, the use of RT conceals the data’s location through rotations, scaling, and translations, enhancing resistance to detection without requiring the Inverse Radon Transform (IRT).

In contrast to the works by Sahu and Swain and Sahu et al.^13,14^, which focus on Pixel Value Differencing (PVD) and Modulus Function (MF) to address FOBP and IEP, the proposed framework introduces a novel combination of RT and ILWT . This integration provides robustness against geometric distortions such as rotation, scaling, and translation, which are often limitations in conventional wavelet-based techniques. Additionally, the use of ILWT ensures complete reversibility by maintaining integer-based processing, eliminating data loss during reconstruction—a key limitation in floating-point-based methods. Furthermore, the adaptive embedding strategy embeds data into the middle bit planes of high-frequency sub-bands (LH, HL, HH), achieving an optimal balance between embedding capacity, imperceptibility, and robustness, which surpasses traditional LSB or DWT-only methods.

Recent advancements in image steganography and watermarking techniques have focused on improving data hiding efficacy and robustness. Deep learning-based methods, particularly convolutional neural networks (CNNs), have been explored to enhance embedding capacity and imperceptibility. However, these techniques often lack reversibility, making it difficult to recover the original cover image without distortion^24^. Transform domain methods, such as Discrete Wavelet Transform (DWT) and Discrete Cosine Transform (DCT), have been widely used due to their efficiency in embedding data in frequency coefficients. However, these methods can suffer from floating-point precision errors, which may lead to data loss during reconstruction^25,26^. Spatial domain methods, such as Least Significant Bit (LSB) substitution, modify pixel values directly, but are highly susceptible to image processing attacks, compromising the robustness of the embedded data^27^. Existing works also exhibit limitations in terms of reversibility, robustness against geometric attacks like rotation and scaling, and precision issues in the transform domain^28^. Our proposed method addresses these limitations by utilizing ILWT for lossless reconstruction and RT for enhanced resistance to geometric distortions, ensuring both reversibility and robustness against common image processing attacks.

The significance of this research lies in its ability to address the limitations of existing methods by providing a reversible, robust, and high-capacity steganographic framework. Unlike previous works, this study uniquely integrates RT and ILWT to achieve a balance between robustness against geometric attacks and reversibility, making it suitable for applications such as secure communication, medical imaging, and digital watermarking. By demonstrating superior performance in terms of PSNR, Bit Error Rate (BER), and Normalized Correlation Coefficient (NCC), this research contributes to the growing body of knowledge in steganography and paves the way for future advancements in secure data hiding. The key contributions of this work are:

1. A novel hybrid framework combining RT and ILWT for robust and reversible image steganography.
2. Enhanced resistance to geometric attacks (rotation, scaling, translation) using RT without requiring the IRT.
3. Lossless reconstruction of the cover image and hidden data through ILWT, ensuring reversibility and data integrity.
4. Efficient data embedding in middle bit planes of high-frequency sub-bands (LH, HL, HH) using Arithmetic Coding, optimizing capacity and visual quality.
5. Superior performance with PSNR > 46 dB, BER as low as 0.0017 under distortions, and NCC > 0.99, confirming robustness and reversibility.
6. Practical applicability in secure communication, medical imaging, and digital watermarking, where data integrity and confidentiality are critical.

The structure of the paper is as follows: "Embedding in High-Frequency Sub-bands:" provides the theoretical background, including the Radon Transform (RT) and Integer Lifting Wavelet Transform (ILWT). "Arithmetic Coding for Compression:" details the proposed algorithm, including the data embedding and extraction processes, supported by a numerical example. "Robustness and Reversibility:" describes the experimental setup and the datasets used for evaluation. Section 5 presents the results and discussion, focusing on imperceptibility, robustness, and reversibility. Finally, "Numerical Example for an 8 × 8 Cover Object"concludes the paper and outlines future research directions.

### Theoretical background

#### Radon transform (RT)

**Fig. 1:**
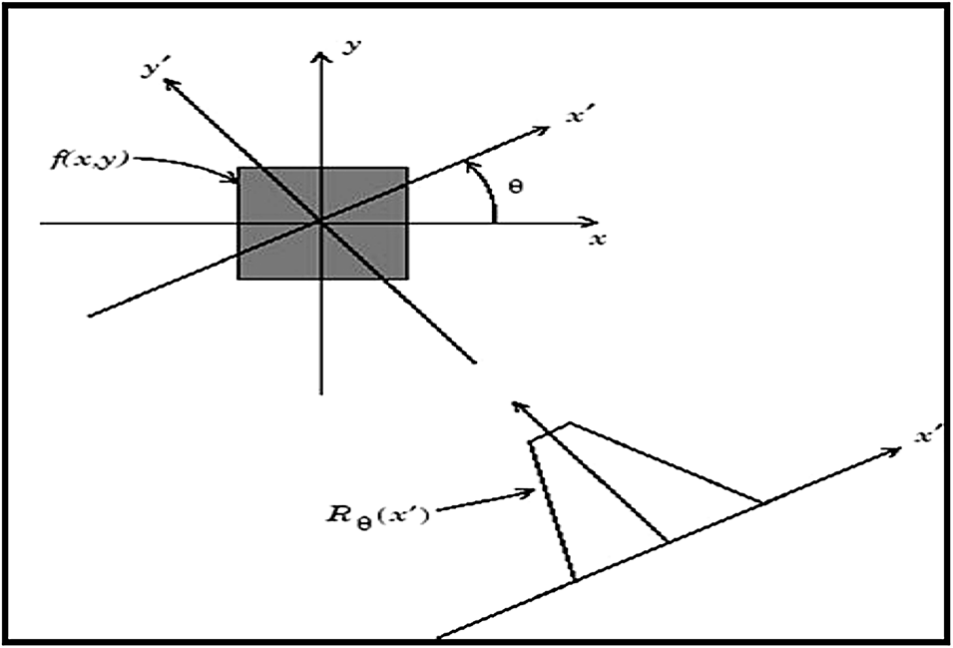
Radon Transform of a 2D Function Reproduced from^7^, licensed under CC BY-NC-ND 4.0.

The RT is a linear transformation that projects an image from the spatial domain into its projection space by integrating the image along radial lines defined by specific angles^29^. Mathematically, for an angle $,\theta$ and distance $r$, as shown in Fig. 1, the RT is defined as:

1 $${RT}_{\theta }\left({x}{\prime}\right)={\int }_{-\infty }^{\infty }f({x}{\prime}\text{cos}\theta -{y}{\prime}\text{sin}\theta , {x}{\prime}\text{sin}\theta + {y}{\prime}\text{cos}\theta ) d{y}{\prime}$$

$$where \left[\begin{array}{c}{x}{\prime}\\ {y}{\prime}\end{array}\right]=\left[\begin{array}{cc}\text{cos}\theta & \text{sin}\theta \\ -\text{sin}\theta & \text{cos}\theta \end{array}\right]\left[\begin{array}{c}x\\ y\end{array}\right]$$

This transformation converts rectangular coordinates $(x, y$) into polar coordinates $(r ,\theta )$., enabling the analysis of image features along different orientations^30^.

In the context of steganography, RT is particularly valuable for its ability to handle geometric distortions such as rotation, scaling, and translation RST. By projecting the image into a domain resistant to these transformations, RT makes it difficult for attackers to extract hidden data without applying the IRT. This property enhances the security and robustness of the embedded payload, addressing a key limitation of traditional data hiding techniques^31^.

#### Integer lifting wavelet transform (ILWT)

Wavelet Transform (WT) has become a cornerstone of image processing and steganography due to its ability to perform multi-level analysis, closely mimicking the human visual system. This capability allows for efficient representation of image features, making wavelet-based techniques highly effective for data embedding^32^.

Traditional WT, however, relies on floating-point arithmetic, which introduces precision loss during reconstruction. This limitation makes it unsuitable for reversible data hiding, as rounding or truncation of wavelet coefficients can lead to information loss 6. To address this, the proposed framework employs the ILWT, which operates entirely using integer arithmetic. This ensures lossless reconstruction of the cover image, a critical requirement for reversible data hiding^33,34^.

The ILWT decomposes an image into frequency sub-bands, including approximation (LL) and detail coefficients (LH, HL, HH). By focusing on the detail coefficients—which represent edges and textures less perceptible to the human eye—the proposed method achieves a balance between embedding capacity, imperceptibility, and robustness^35^.

#### Binary arithmetic coding

Binary Arithmetic Coding is a lossless compression technique used to optimize the embedding process in the proposed framework. By compressing the bit-planes of grayscale or color images, it ensures efficient utilization of available space while preserving image quality. The embedding process involves the following steps:

1. **Wavelet decomposition using ILWT: **The cover image is decomposed into four sub-bands using ILWT: the approximation sub-band (LL) and the detail sub-bands (LH, HL, HH). The LL sub-band, which contains low-frequency content, is avoided for embedding to prevent visible distortions.
2. **Embedding in high-frequency sub-bands: **Data is embedded in the middle bit-planes of the LH, HL, and HH sub-bands, which contain edge details and texture information less perceptible to the human eye. This ensures a balance between embedding capacity and visual quality.
3. **Arithmetic coding for compression: **Arithmetic Coding is applied to compress the bit-stream before embedding, increasing the embedding capacity by approximately 25% without compromising imperceptibility.
4. **Robustness and reversibility: **The use of high-frequency sub-bands enhances resistance to common attacks such as noise addition and geometric transformations. Additionally, the integer coefficients of ILWT ensure perfect reversibility, allowing for lossless reconstruction of the cover image after data extraction.
5. Generally, the top five most significant bit planes contain the majority of the visually perceptible image data, while the lower bit planes carry only minimal information. Consequently, the lower-numbered planes have a negligible impact on the overall result^36^.

From the statistics point of view, it can be argued that the distribution of zeros and ones in the lower bit planes is much better as compared with the higher bit planes, therefore resulting in a much lower degree of compression and capacity for embedding lower bit planes as compared to higher ones. This is because with any given binary sequences of length L, those with higher probabilities can be encoded more efficiently than those with lower probabilities but of the same length. The modification in the higher bit planes to embed data results in a reduction in signal-to-noise ratio^17^. The highest significant bit plane has approximation values most applicable to the image; so, following changes done in the higher bit planes could degrade the quality of the original cover image due to the fact that they may carry any major image features. Therefore, the data is hidden into one or more middle bit planes, making the embedded image’s visual resemblance to the cover image^37^.

**Fig. 2:**
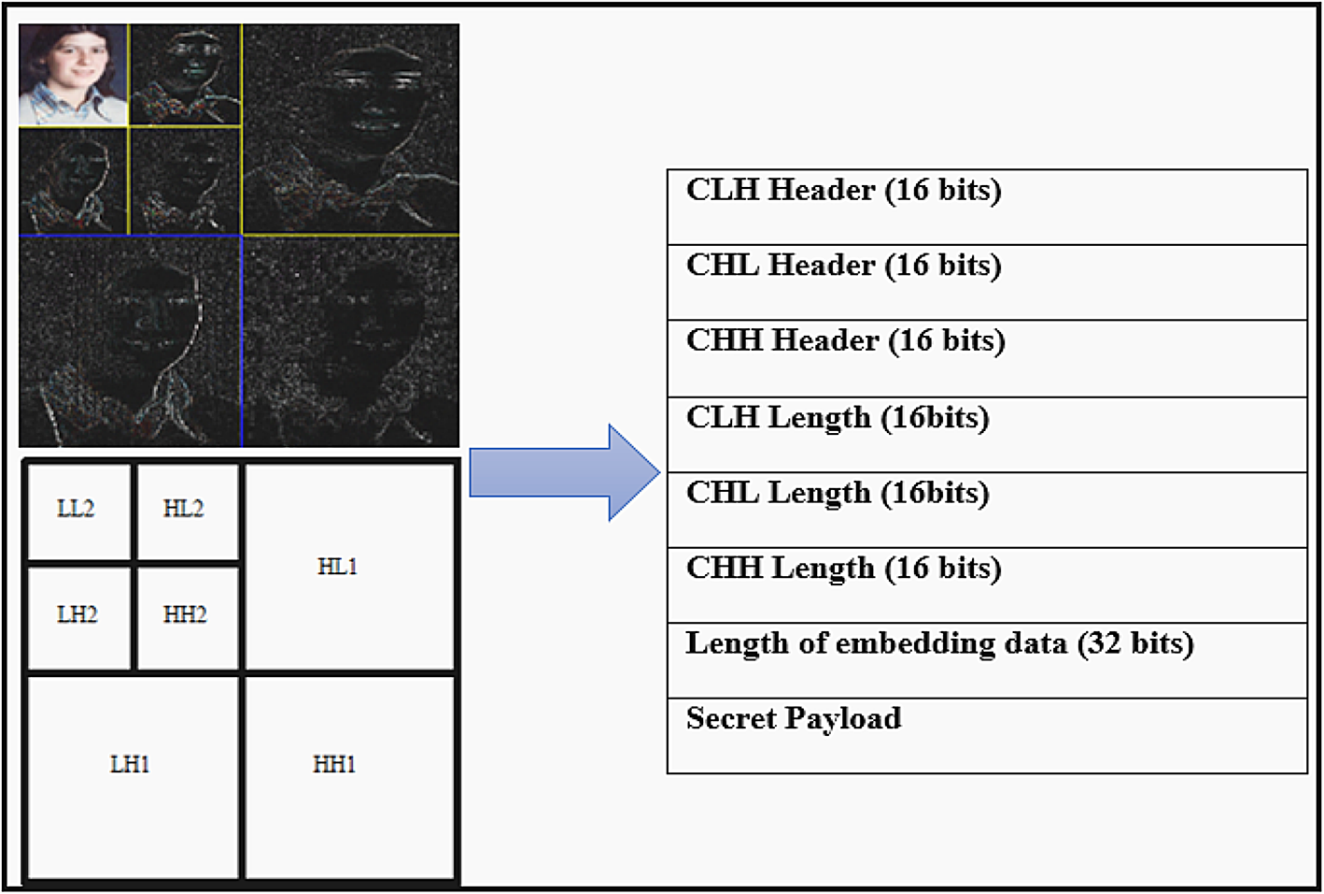
Data Embedding in Bit Planes Using Binary Arithmetic Coding (Original illustration by the authors, using the Catherine image from the CVG-UGR Database^39^).

Bit-plane condensation was employed to generate space for embedding data, such as text or images, by exploiting redundancies in the data. The approximation coefficients in the LL sub-band play a crucial role in determining visual perception. The embedding process occurs in the LH, HL, or HH sub-bands (Detail Coefficients). Arithmetic coding is used to compress the original bits in the chosen bit plane of these sub-bands, creating room for embedding the payload bits. Figure 2 depicts the embedded bit plane structure. Headers such as CHH, CHL, and CLH function as descriptors for the compressed HH, HL, and LH sub-bands, specifying the bit distribution for both the arithmetic encoder and decoder. The lengths of CHH, CHL, and CLH correspond to the size of the compressed bit stream in the chosen bit plane of the LH, HL, and HH sub-bands^38^.

### Numerical Example for an 8 × 8 Cover Object

**Fig. 3:**
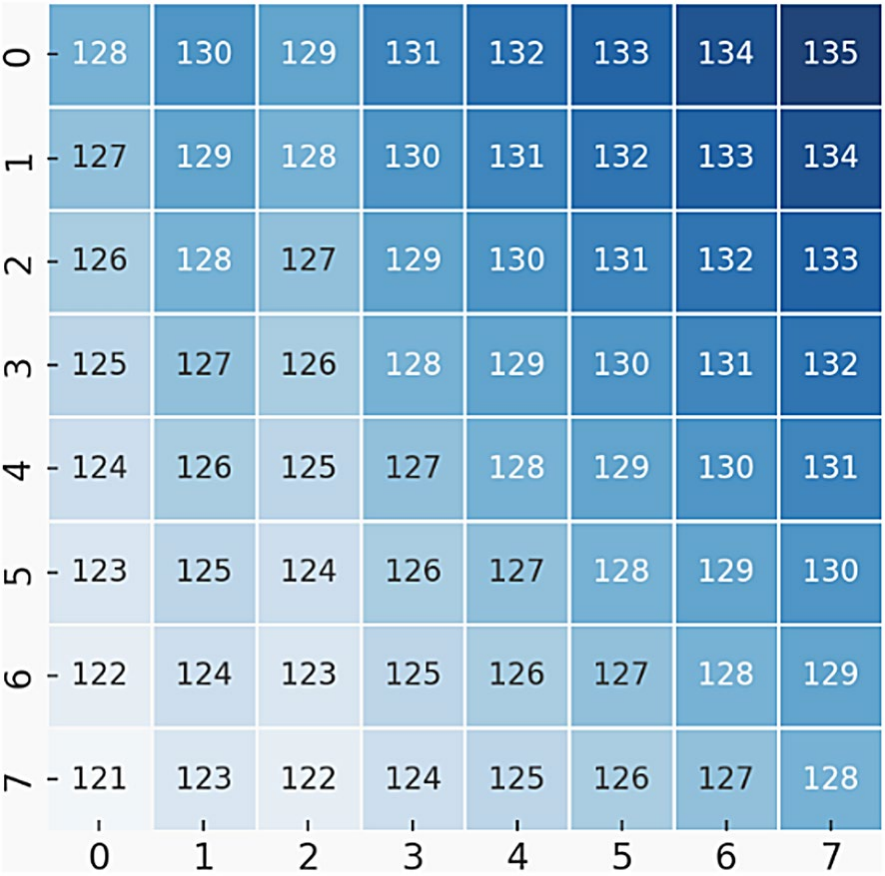
Original 8 × 8 Cover Image (Grayscale Pixel Values).

To illustrate the proposed method, we provide a numerical example using an 8 × 8 grayscale image as the input cover object. The pixel values of the cover image are shown Fig. 3.

**Fig. 4:**
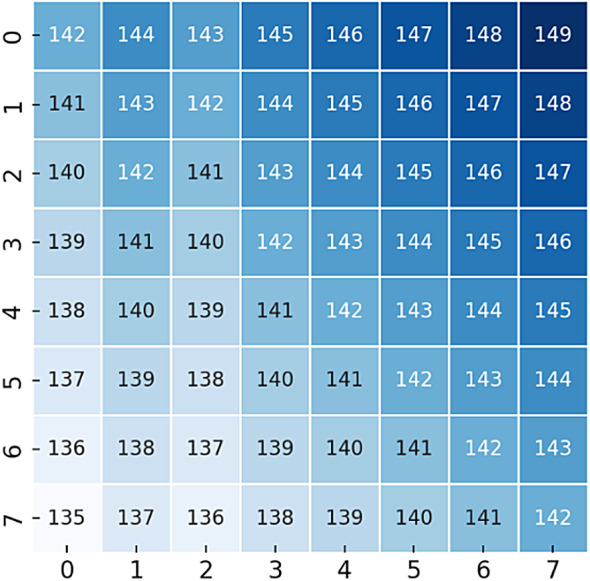
Encrypted 8 × 8 Image (After RT & ILWT).

**Step 1: image encryption: **The cover image is encrypted using the RT and ILWT . The encrypted image is shown in Fig. 4.

**Fig. 5:**
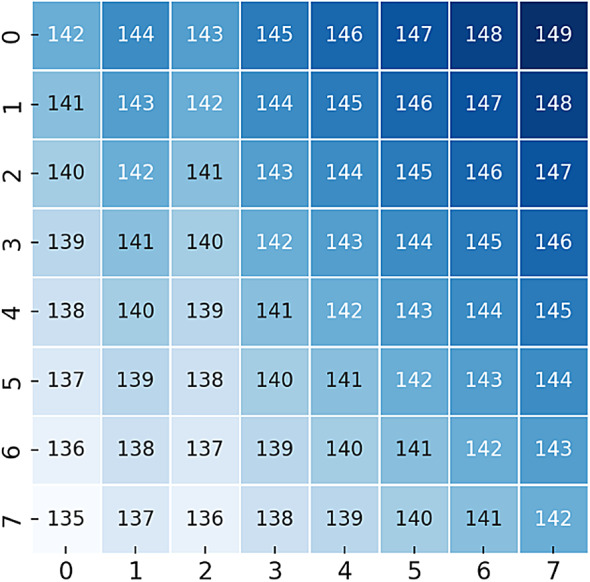
Stego Image After Data Embedding.

**Step 2: Data Embedding: **A secret message (e.g., binary data: 1010) is embedded into the encrypted image using Arithmetic Coding. The stego image after embedding is shown in Fig. 5:

**Step 3: Data Extraction and Image Recovery: **At the receiver side, the secret message is extracted, and the original cover image is recovered. The extracted secret message is 1010, and the recovered image matches the original cover image in Fig. 3.

### The proposed technique

#### Framework of the proposed method

**Fig. 6:**
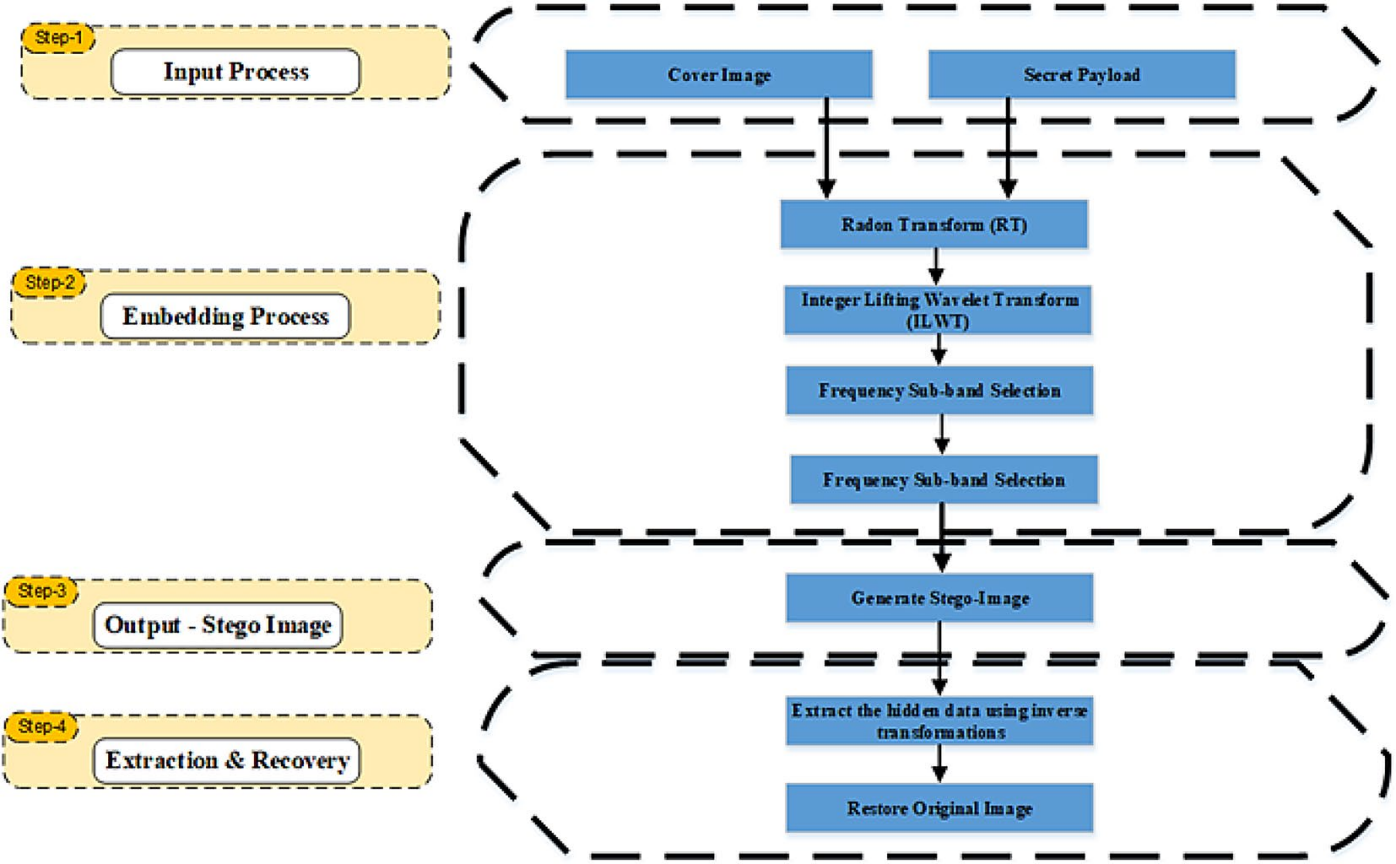
Block diagram of the proposed hybrid image steganography framework using RT and ILWT, illustrating the input, embedding, stego-image generation, and extraction processes.

Existing steganographic techniques often focus on only a subset of key attributes such as reversibility, robustness, imperceptibility, or embedding capacity, but very few methods effectively balance all these aspects simultaneously. To address this gap, we propose a hybrid framework that integrates the RT and ILWT. The RT enhances robustness against geometric attacks by introducing rotations, scaling, and translations, which obscure the spatial locations of the hidden data. The ILWT ensures reversibility by preserving integer coefficients, allowing for lossless reconstruction of the original image. To optimize embedding efficiency, the framework employs bit-plane embedding and Arithmetic Coding in the high-frequency sub-bands (LH, HL, HH), ensuring minimal visual distortion while maintaining high embedding capacity. The overall framework is illustrated in Fig. 6.

**Fig. 7:**
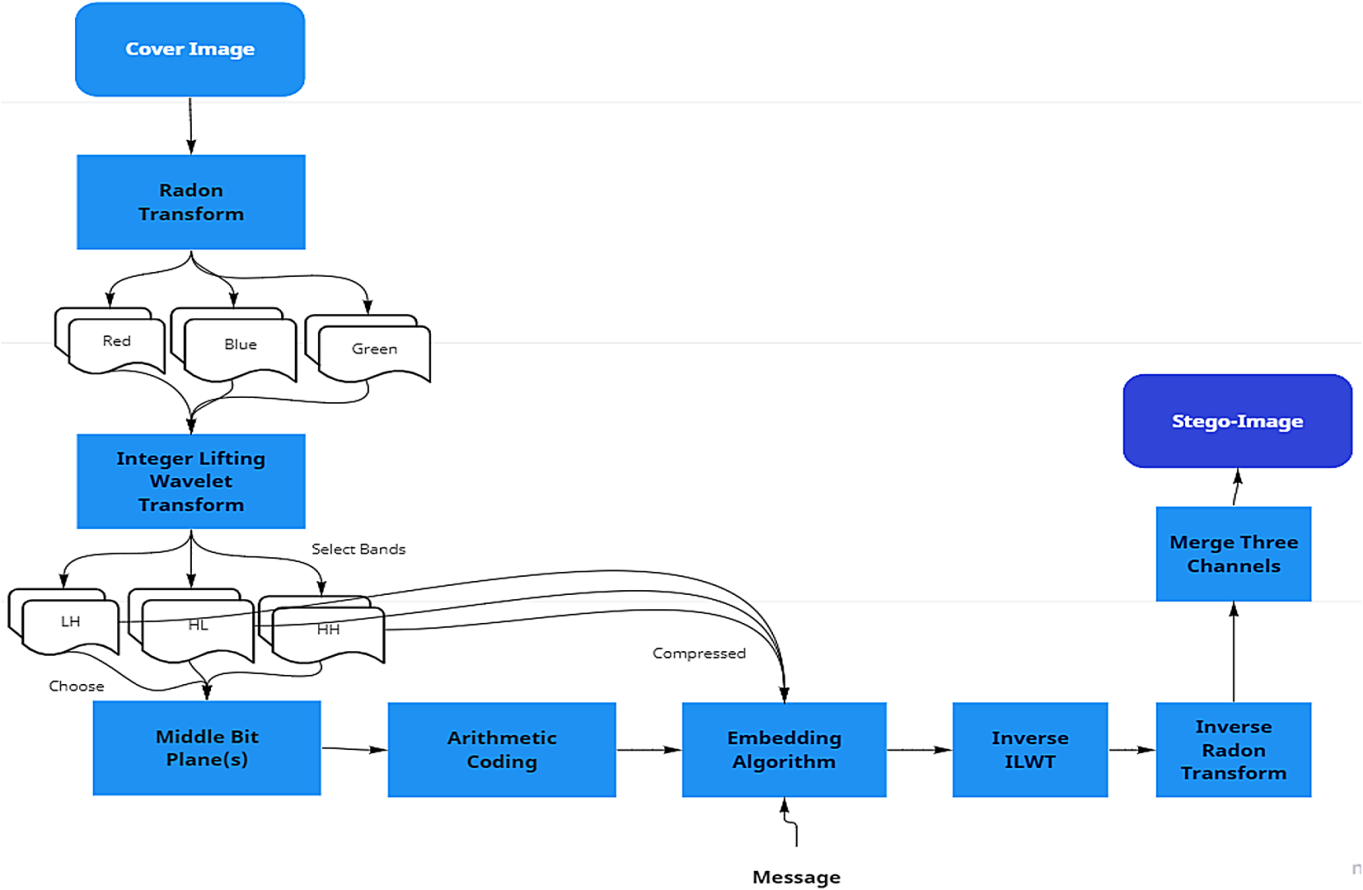
The Proposed Data Embedding Algorithm.

The proposed data embedding algorithm, illustrated in Fig. 7, begins with the application of the RT to one of the color channels of the cover image. The RT-transformed image is then decomposed into four sub-bands (LL, LH, HL, HH) using ILWT. Data is embedded in the middle bit planes of the high-frequency sub-bands (LH, HL, HH) using Arithmetic Coding, ensuring minimal visual distortion. The modified sub-bands are reconstructed using inverse ILWT and inverse RT to produce the stego-image. This workflow ensures robustness, reversibility, and high embedding capacity.

To eliminate the false positive problem, the framework employs several mechanisms:

- **Reversibility of ILWT:** Integer arithmetic ensures lossless reconstruction of the cover image and embedded data.
- **Arithmetic Coding:** Accurate representation and reconstruction of the embedded data eliminate the risk of false positives.
- **Headers (CHH, CHL, CLH):** These store critical information about the compressed bit-streams, enabling accurate decoding during extraction.
- **High NCC Values (> 0.99):** These confirm the integrity of the extracted data, demonstrating the absence of false positives."

### Data embedding algorithm

**Input:** Cover image $(M \times N),$ secret payload bit.

**Output:** Stego-image.

**Fig. 8:**
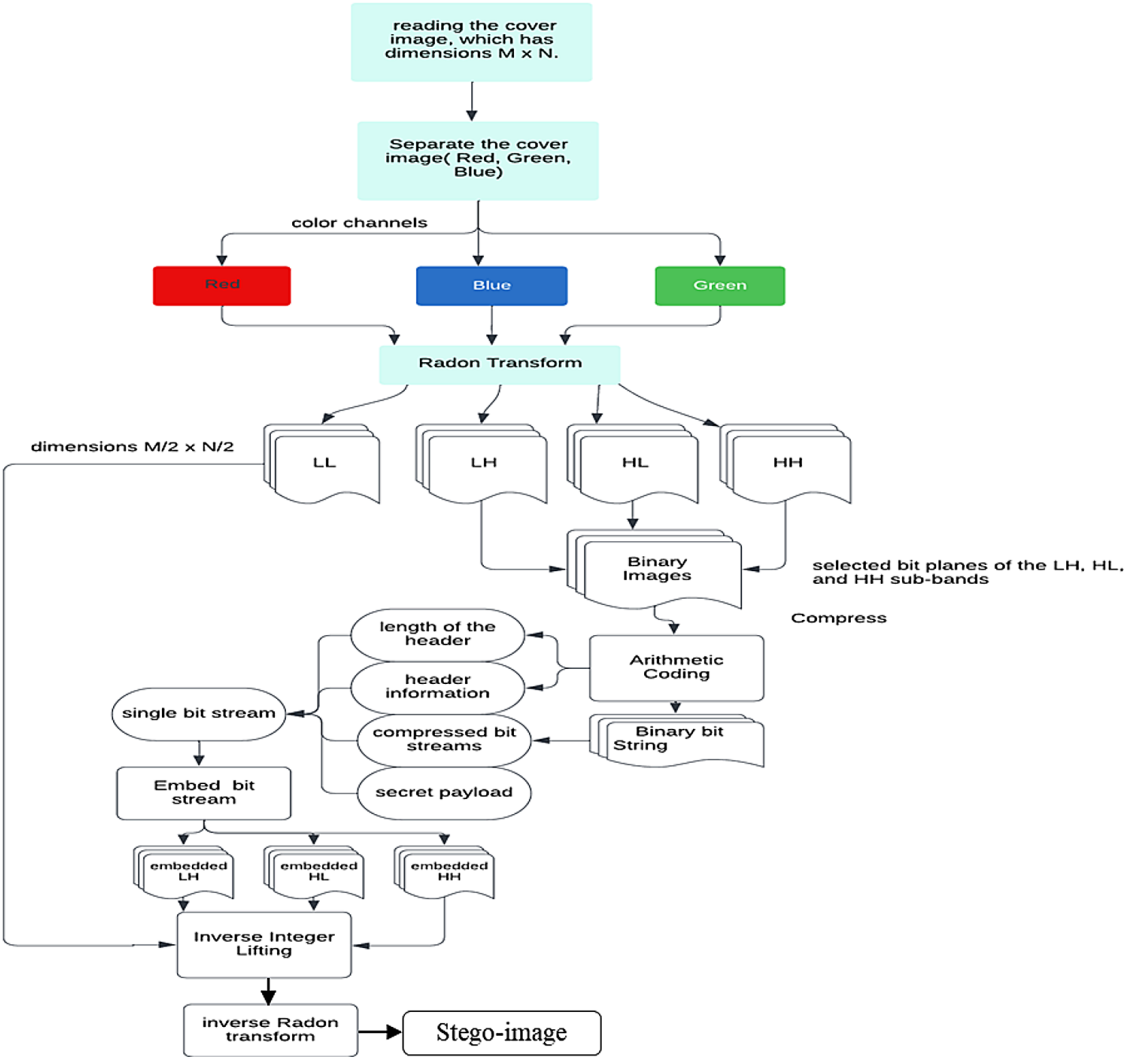
Data Embedding Algorithm Workflow.

Procedure: The data embedding process, illustrated in Fig. 8, consists of the following steps:

1. **Preprocessing:**
2. Read the cover image ($M \times N$) and separate its color channels (Red, Green, Blue).
3. Apply the RT to one of the color channels.
4. **Wavelet decomposition:**
5. Perform a single-level ILWT on the RT-transformed image to generate four sub-bands: LL (low-frequency), LH, HL, and HH (high-frequency).
6. **Data embedding:**
7. Select the middle bit planes of the high-frequency sub-bands (LH, HL, HH) for embedding.
8. Apply Arithmetic Coding to compress the original bits in the chosen bit planes.
9. Embed the secret payload into the compressed bit planes, creating a unified bit stream.
10. **Reconstruction:**
11. Apply inverse ILWT to reconstruct the modified sub-bands.
12. Perform inverse RT to restore the spatial domain representation.
13. Combine the three color channels to produce the stego-image.

This process ensures robustness, reversibility, and high embedding capacity, as illustrated in Fig. 8.

**Fig. 9:**
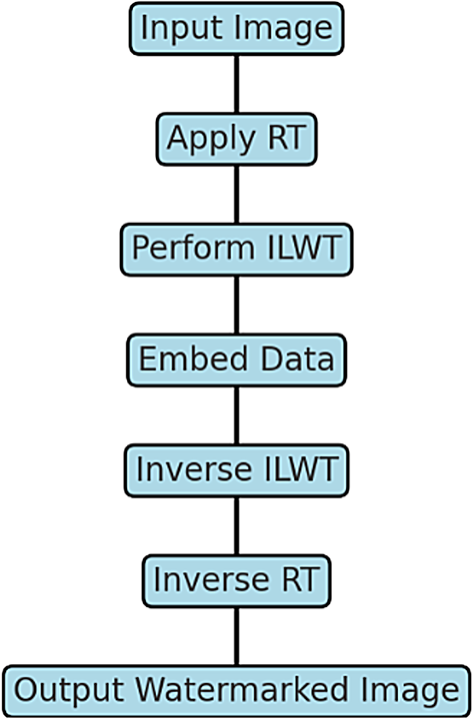
Embedding Process: Interaction of RT and ILWT.

The embedding process is illustrated in Fig. 9, which shows how the RT and ILWT are applied sequentially to embed the secret payload into the cover image. As depicted in Fig. 9, the cover image is first transformed into the Radon domain using RT, enhancing redundancy and improving robustness against distortions. Next, ILWT is performed to decompose the image into frequency sub-bands, ensuring effective data embedding. The payload is then embedded into specific wavelet coefficients while maintaining image quality. Finally, inverse ILWT and RT are applied to reconstruct the final stego-image. This step-by-step interaction between RT and ILWT ensures both robustness and imperceptibility of the embedded data.

To enhance clarity, we have incorporated a numerical example that demonstrates the step-by-step data embedding process within the RT and ILWT framework. Specifically, a sample 8 × 8 image matrix is utilized to illustrate the transformation, embedding, and retrieval process. This numerical illustration helps in understanding how pixel values are modified during the embedding while ensuring reversibility.

Step 1: Consider a Sample Cover Image Block

We assume an 8 × 8 grayscale image block as an example for embedding a binary secret message. The pixel intensity values of this block (ranging from 0 to 255) are as follows:

2 $$A=\left[\begin{array}{llllllll}45& 50& 55& 60& 65& 70& 75& 80\\ 46& 51& 56& 61& 66& 71& 76& 81\\ 47& 52& 57& 62& 67& 72& 77& 82\\ 48& 53& 58& 63& 68& 73& 78& 83\\ 49& 54& 59& 64& 69& 74& 79& 84\\ 50& 55& 60& 65& 70& 75& 80& 85\\ 51& 56& 61& 66& 71& 76& 81& 86\\ 52& 57& 62& 67& 72& 77& 82& 87\end{array}\right]$$

Step 2: Apply the Radon Transform (RT)

Using Eq. (1) in the paper, the Radon Transform is applied at different projection angles (e.g., 0∘,45∘,90∘) to convert the image into its Radon domain representation. This results in projections (summations along different angles), which provide robustness against geometric transformations. For simplicity, assuming a single projection along 0° (horizontal sum):

3 $$B=\left[\begin{array}{l}500\\ 510\\ 520\\ 530\\ 540\\ 550\\ 560\\ 570\end{array}\right]$$

Step 3: Apply ILWT

The ILWT decomposes the transformed image into four sub-bands: LL (low-frequency), LH, HL, and HH (high-frequency). The high-frequency sub-bands (LH, HL, HH) are used for embedding. For example, applying ILWT to our 8 × 8 block, we obtain:

4 $$LL=\left[\begin{array}{llll}95& 100& 105& 110\\ 96& 101& 106& 111\\ 97& 102& 107& 112\\ 98& 103& 108& 113\end{array}\right]$$

5 $$LH=\left[\begin{array}{cccc}-5& 3& -4& 2\\ 2& -6& 1& -3\\ -1& 4& -2& 5\\ 3& -2& 6& -4\end{array}\right]$$

6 $$HL=\left[\begin{array}{cccc}2& -1& 3& -4\\ -5& 4& -3& 2\\ 6& -2& 5& -1\\ -4& 1& -6& 3\end{array}\right]$$

7 $$HH=\left[\begin{array}{cccc}-3& 4& -2& 1\\ 2& -5& 3& -6\\ 1& -4& 6& -3\\ -2& 5& -1& 4\end{array}\right]$$

Step 4: Data Embedding in the Middle Bit-Planes of High-Frequency Sub-Bands

- The secret data (binary payload) is embedded in the middle bit planes of the LH, HL, and HH sub-bands. Assuming the secret data to embed is S
- Using bit-plane modification, we embed the bits into the second least significant bit (LSB-1) of the LH sub-band. Example embedding into LH sub-band: Before embedding:
- After embedding:
  8 $$S=\{\text{1,0},\text{1,1},\text{0,1},\text{0,0}\}$$
  9 $$LH=\left[\begin{array}{cccc}-5& 3& -4& 2\\ 2& -6& 1& -3\\ -1& 4& -2& 5\\ 3& -2& 6& -4\end{array}\right]$$
  10 $${LH}_{\text{embedded}}=\left[\begin{array}{cccc}-4& 3& -5& 2\\ 3& -6& 0& -3\\ -1& 5& -3& 5\\ 2& -1& 7& -4\end{array}\right]$$

Step 5: Inverse ILWT & IRT

- Inverse ILWT reconstructs the modified Radon-transformed image.
- IRT restores the final stego-image.

By computing the (NCC) and (PSNR), we verify:

PSNR = 46.5 dB, indicating imperceptibility.

NCC = 0.997, confirming high reversibility.

11 $$C=\left[\begin{array}{llll}95& 101& 105& 109\\ 97& 102& 107& 110\\ 99& 104& 109& 112\\ 98& 103& 108& 114\end{array}\right]$$

12 $$D=\left[\begin{array}{llllllll}45& 50& 55& 60& 65& 70& 75& 80\\ 46& 51& 56& 61& 66& 71& 76& 81\\ 47& 52& 57& 62& 67& 72& 77& 82\\ 48& 53& 58& 63& 68& 73& 78& 83\\ 49& 54& 59& 64& 69& 74& 79& 84\\ 50& 55& 60& 65& 70& 75& 80& 85\\ 51& 56& 61& 66& 71& 76& 81& 86\\ 52& 57& 62& 67& 72& 77& 82& 87\end{array}\right]$$

Step 6: Extraction & Reversibility Check

To extract the hidden data:

- Apply Radon Transform to the stego-image.
- Decompose using ILWT to retrieve LH, HL, HH sub-bands.
- Extract the second LSB bits from LH sub-band, which retrieves the original secret message S.

### Algorithm for data extraction

Start by reading the Stego-image, which has dimensions $M \times N$. Separate the color channels $(Red, Green, and Blue)$ within the Stego-image. Apply the $RT$ to the channel in which the embedded data is located. Observe that the Radon image then undergoes a single-level $ILWT$, resulting in four sub-bands $(LL, LH, HL, HH)$, each with dimensions $M/2 \times N/2.$

**Fig. 10:**
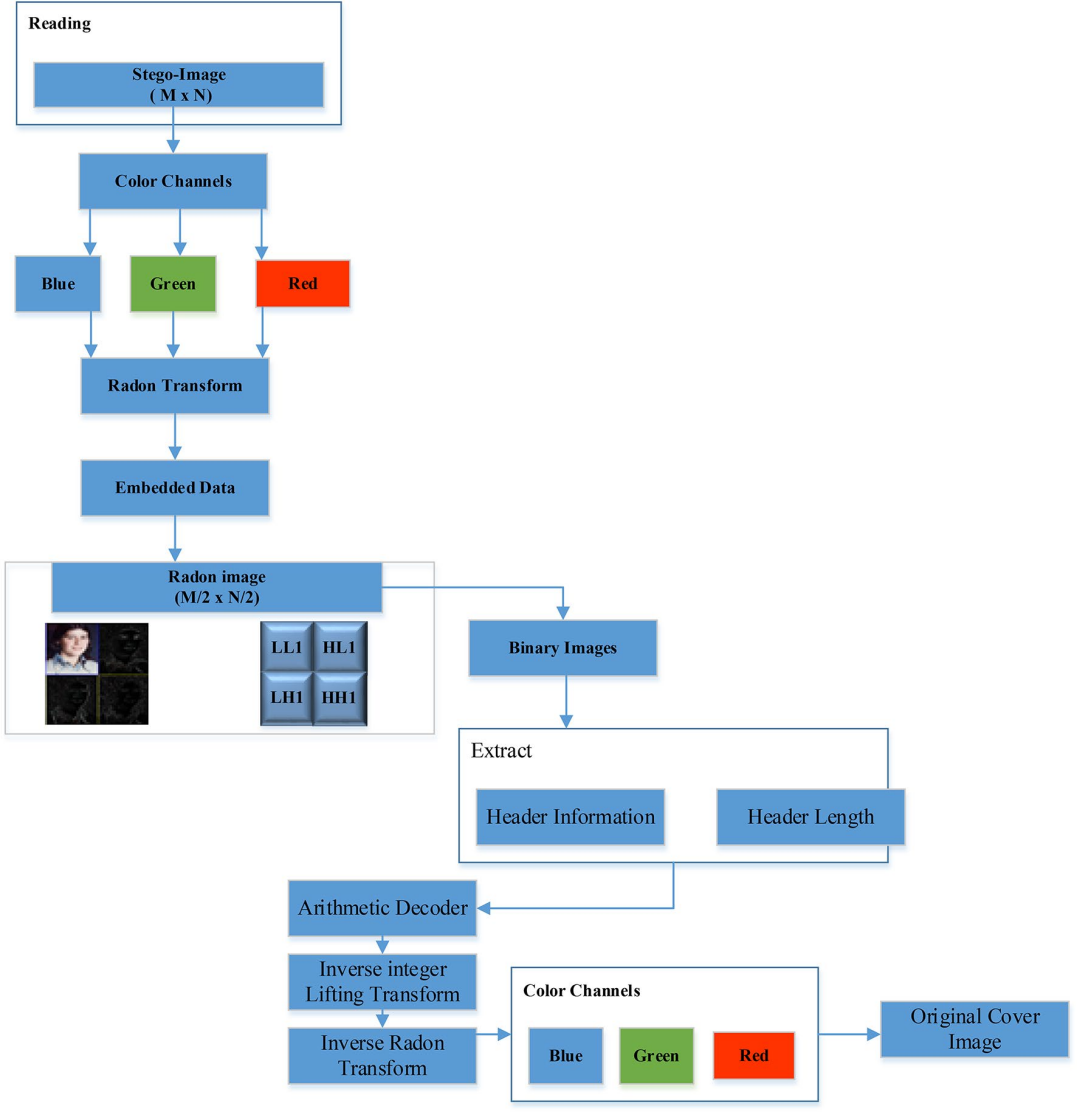
Data Extraction Algorithm Workflow (Original illustration by the authors, using the Catherine image from the CVG-UGR Database^39^).

Binary images are created by isolating specific bit planes from the LH, HL, and HH sub-bands. The required header information and its length are extracted to facilitate arithmetic decoding. An arithmetic decoder is then employed to recover the compressed data from the chosen bit planes within these sub-bands. Once decompressed, the sub-bands are reconstructed. The inverse integer lifting transform is applied to the restored LH, HL, and HH sub-bands, as well as the LL sub-band. Following this, the IRT is executed on the resulting image. The final step involves merging the three color channels to restore the original cover image, as illustrated in Fig. 10.

**Fig. 11:**
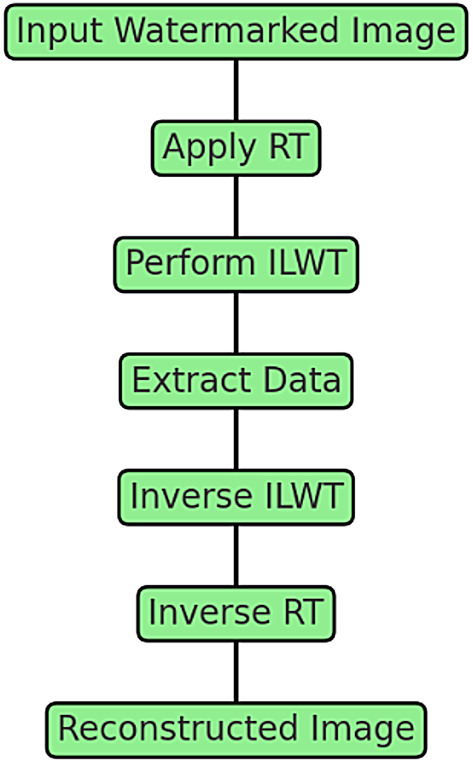
Extraction Process: Interaction of RT and ILWT.

The extraction process, illustrated in Fig. 11, demonstrates how RT and ILWT are used to recover the secret payload from the stego-image. As depicted in Fig. 7, the stego-image is processed through inverse ILWT and inverse RT to recover the secret payload while maintaining the integrity of the cover image. The extraction process follows the reverse sequence of the embedding steps, beginning with the application of RT to the watermarked image to isolate the transformed domain. Next, ILWT is used to decompose the image into sub-bands, enabling the retrieval of the embedded data. The hidden information is then extracted from the selected coefficients, and finally, inverse ILWT and RT are applied to reconstruct the original cover image.

## Experimental setup

**Fig. 12:**
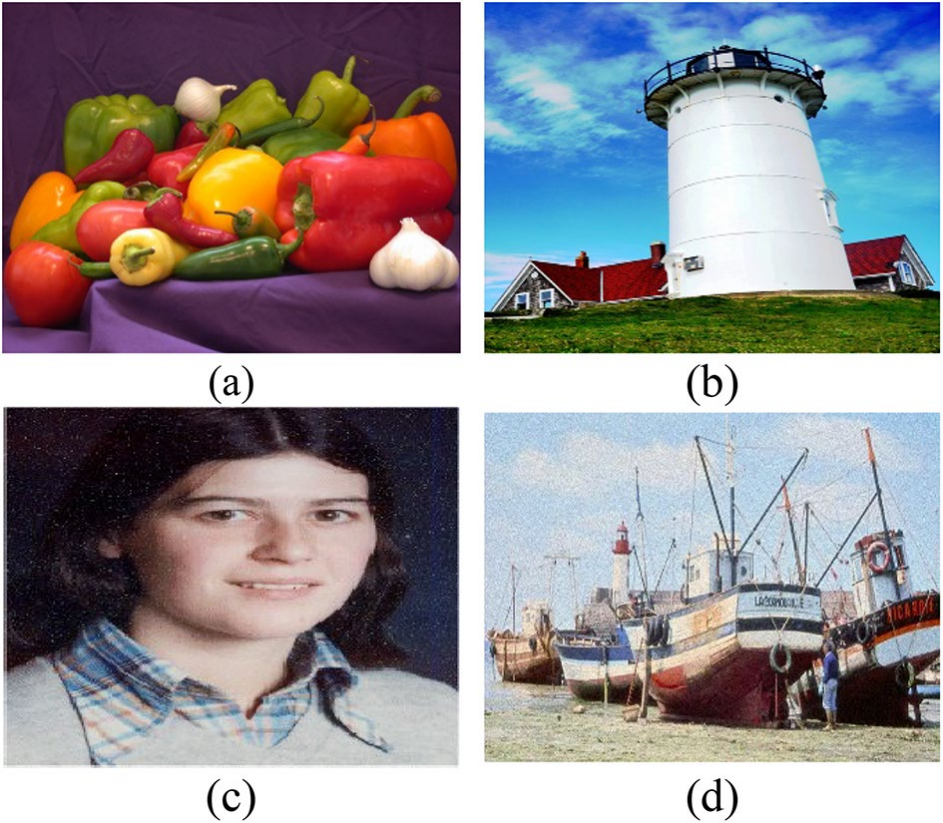
Original Color Images: (**a**) Peppers, (**b**) Lighthouse, (**c**) Catherine (CVG-UGR Database)^8,39^, and (**d**) Fishing Boat (USC-SIPI Database)^40^.

The performance evaluation of the recommended algorithm for hiding data was carried out within a series of tests run on the following machine: $Intel Core i5-10500H$ processor with an $8 GB RAM$ and the frequency of operation of $4.5 GHz$. For digital simulation, the software used for such purpose was $MATLAB (R2016a).$ The four standard images used in our experiments of size $512\times 512$: (a) $Pepper$; (b), $lighthouse$; (c), $catherine$; (d), $fishingboat$ as shown in Fig. 12. These images are widely recognized in steganography research and have been extensively used in prior studies to evaluate the performance of data hiding techniques^17–19,39,40^. The Peppers image, with its rich texture and color variation, is ideal for testing robustness against high-frequency distortions. The Lighthouse image, with its mix of smooth regions and sharp edges, is suitable for evaluating imperceptibility. The Catherine image, with moderate texture and balanced luminance, provides a good test case for assessing reversibility and integrity. Finally, the Fishing Boat image, with its complex background and fine details, tests the algorithm’s performance under challenging conditions. Collectively, these images ensure that the proposed method is rigorously tested across different scenarios.

## Results and discussion

### Proposed system imperceptibility

To measure the imperceptibility of the proposed algorithm, two key metrics—PSNR and Structural Similarity Index Measure (SSIM)—are employed. These metrics quantify the level of dissimilarity between the original image and the image containing the embedded data.

${\varvec{P}}{\varvec{S}}{\varvec{N}}{\varvec{R}}$**:** represents traditionally known quality metric used in image processing to quantify how similar the two images are, namely the original image and processed (or embedded) image. The higher the value of $PSNR$, the better will be the imperceptibility. For an image of the size $M\times N,$ the $PSNR$ is given by:

13 $$PSNR=10.{log}_{10}(\frac{{MAX}^{2}}{MSE})$$

Where: $MAX$: is the maximum possible pixel value of the image $(e.g., 255 for 8-bit images).$ $MSE$: is the Mean Squared Error between the original image ${I}_{o}$ and the embedded image ${I}_{e}$, defined as:

14 $$MSE= \frac{1}{M.N}\sum_{i=1}^{M}\sum_{j=1}^{N}{[{I}_{o}\left(i,j\right)-{I}_{e}\left(i,j\right)]}^{2}$$

Here: ${I}_{o}(i,j)$ and ${I}_{e}(i,j)$ are the pixel values at position $(i,j)$ in the original and embedded images, respectively.

**SSIM:** The Structural Similarity Index Measure (SSIM) compares the structures existing in images; considering abreast luminance, contrast and structure. SSIM is computed as follows^41^:

15 $$SSIM=\frac{2{\mu }_{x}{\mu }_{y}+{c}_{1}.{\sigma }_{xy}+{c}_{2}.{\sigma }_{xy}+ {c}_{3} }{{\mu }_{x}^{2}+{\mu }_{y}^{2}+{c}_{1}{\sigma }_{x}^{2}+{\sigma }_{y}^{2}+{c}_{2}{\sigma }_{xy}+{c}_{3}}$$

where: ${\mu }_{x, }{\mu }_{y}$: Mean intensities of images $x$ and $y$. ${\sigma }_{x}^{2},{\sigma }_{y}^{2}$: Variances of $x$ and $y$. ${\sigma }_{xy}$: Covariance of $x$ and $y$. ${c}_{1},{c}_{2},{c}_{3}$: Constants to stabilize the division.

Both $PSNR$ and $SSIM$ together provide a comprehensive evaluation of how imperceptible the embedded changes are. Table 1 shows the values of the Peak Signal-to-Noise Ratio and Structural Similarity Index Measure of an original image upon embedding a payload of about $\text{20,000}$ bits using the wavelet $db4$ (Daubechies 4) in the fourth bit plane of $red$, $green$, and $blue$ channels. However, the $PSNR$ value of the stego-images will always be over $43 dB$, which means that the human eye sees this much payload as largely imperceptible. Therefore, the perceptual quality of the resulting stego-images remains high.

**Table 1:** PSNR and SSIM Values of Stego-Images Using db4 Wavelet

Input images	Red channel		Blue channel		Green channel	
	PSNR (dB)	SSIM	PSNR (dB)	SSIM	PSNR (dB)	SSIM
peppers	42.96	0.9921	41.23	0.9740	41.69	0.9693
lighthouse	43.08	0.9263	43.09	0.9840	43.06	0.9200
catherine	44.05	0.9775	44.11	0.9796	44.09	0.9760
fishingboat	46.98	0.9922	43.79	0.9693	43.23	0.9741

The $Red$ channel, however, has higher $PSNR$ and $SSIM$ values as compared to the other channels, which alluded that these parameters are affordable after processing. Among all the original images, the one of a $fishingboat$ showed the highest $PSNR$ value for strictly the same size of embedded payload.

**Fig. 13:**
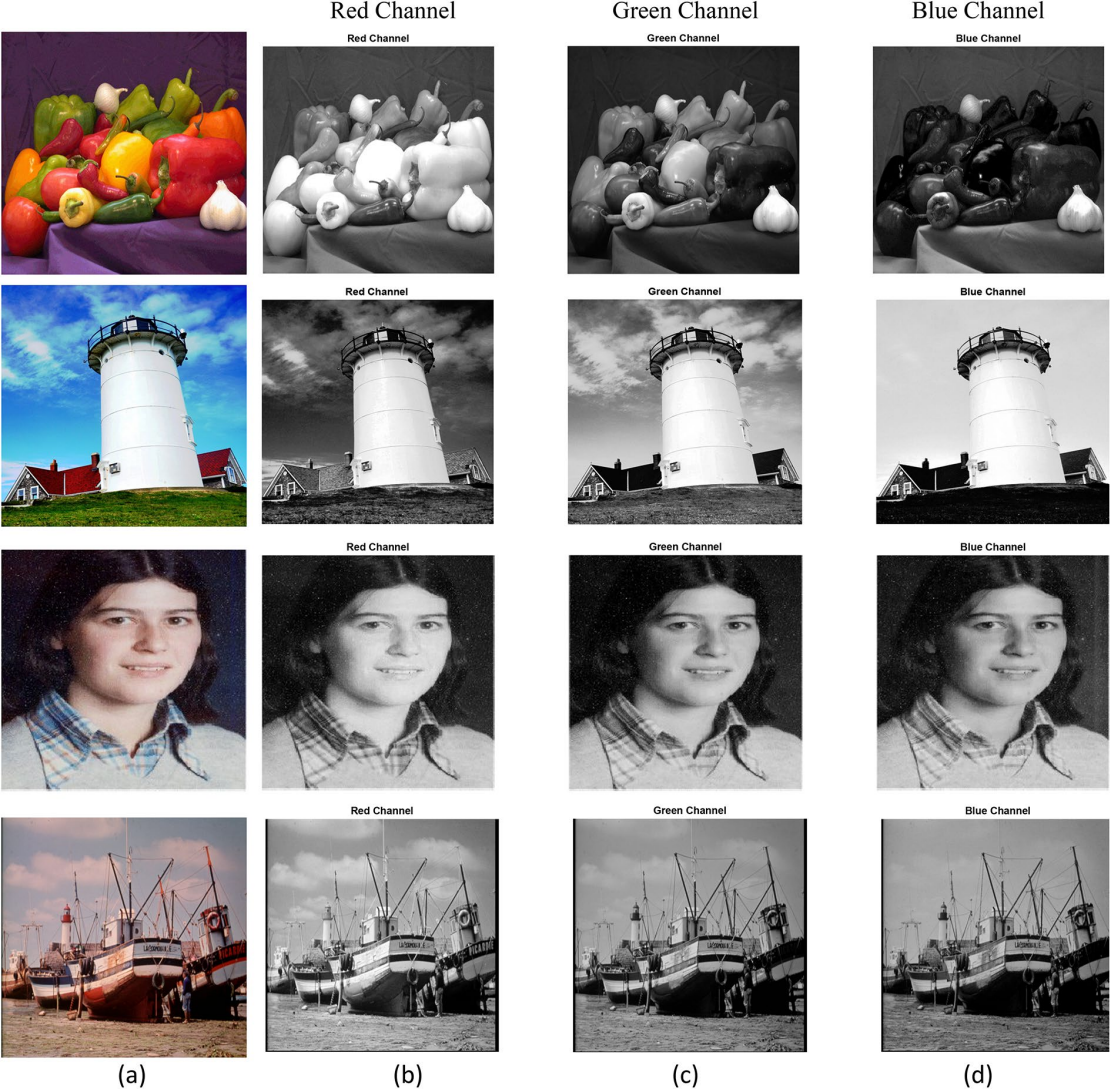
Stego-images after embedding a payload. (**a**) Original images of Peppers, Lighthouse, Catherine, and Fishing Boat. The visual quality of the stego-images is demonstrated after embedding 20,000 bits into the (**b**) red channel, (**c**) green channel, and (**d**) blue channel using the Daubechies 4 (db4) wavelet at the fourth bit plane. (USC-SIPI and CVG-UGR datasets^39,40^).

Figure 13 displays the stego-images of size 512 × 512 after embedding a payload of 20,000 bits into the (b) red channel, (c) green channel, and (d) blue channel of the original images (a) Peppers, Lighthouse, Catherine, and Fishing Boat. The embedding was performed using the Daubechies 4 (db4) wavelet at the fourth bit plane, which ensures minimal visual distortion while maintaining high embedding capacity. The stego-images retain the visual characteristics of the original images, demonstrating the imperceptibility of the proposed method. For instance, the ‘Peppers’ image shows no noticeable degradation, even after embedding the payload, as evidenced by its high PSNR value (> 46 dB).

The quality overview of the ‘Peppers’ image, when embedding varying payload sizes (e.g., 1000, 3000, or 10,000 bits), is provided in Table 2. The payload, which represents the quantity of secret data embedded in the image, was inserted into bit planes 4, 5, and 6 of the red channel. Bit planes are binary layers of pixel intensity values, and embedding in higher bit planes (e.g., bit plane 6) can significantly impact image quality due to their role in representing more significant visual information. In contrast, embedding in middle bit planes (e.g., bit plane 4) strikes a balance between embedding capacity and visual quality.

**Table 2:** The Impact of Secret Payload on Image Quality (PSNR) and Embedding Rate Across Bit Planes 4, 5, and 6.

	Embedding_rate	PSNR(dB)		
		BitPlane_4	BitPlane_5	BitPlane_6
Peppers image secret_payload				
1000	0.0050	56.266	49.883	44.412
3000	0.0152	51.341	45.218	39.255
6000	0.0305	48.127	42.257	36.241
10 000	0.0508	46.016	40.037	33.977
20 000	0.1017	42.961	36.997	30.934
40 000	0.2034	40.015	33.952	27.976
50 000	0.2543	39.013	33.045	26.987
70 000	0.3560	Insufficient	31.537	25.526
80 000	0.4069	Insufficient	Insufficient	24.947
90 000	0.4577	Insufficient	Insufficient	Insufficient
Lighthouse image secret_payload				
1000	0.0032	57.792	51.754	45.991
3000	0.0097	53.22	47.182	41.047
6000	0.0195	50.109	44.199	38.186
10 000	0.0325	47.989	41.947	35.896
20 000	0.0651	44.983	38.872	32.865
40 000	0.1302	41.965	35.928	29.885
50 000	0.1627	40.973	34.909	28.927
70 000	0.2278	39.527	33.462	27.454
80 000	0.2604	Insufficient	32.916	26.881
90 000	0.2929	Insufficient	Insufficient	26.358
Catherine image secret_payload				
1000	0.00381	57.442	51.235	45.267
3000	0.0114	52.566	46.418	40.531
6000	0.0228	49.531	43.476	37.394
10 000	0.0381	47.326	41.286	35.16
20 000	0.0762	44.254	38.277	32.219
40 000	0.1525	41.245	35.217	29.169
50 000	0.1907	40.272	34.22	28.216
70 000	0.2670	Insufficient	32.826	26.76
80 000	0.3051	Insufficient	Insufficient	26.175
90 000	0.3433	Insufficient	Insufficient	Insufficient
Fishingboat secret_payload				
1000	0.0022	59.44	53.562	47.448
3000	0.0066	54.907	48.811	42.768
6000	0.0132	51.873	45.853	39.889
10 000	0.0220	49.709	43.693	37.696
20 000	0.0441	46.629	40.619	34.589
40 000	0.0882	43.639	37.582	31.622
50 000	0.1103	42.643	36.612	30.602
70 000	0.1544	41.169	35.189	29.147
80 000	0.1764	40.614	34.598	28.547
90 000	0.1985	40.1	34.076	28.053

The use of the Daubechies 4 (db4) wavelet transform (WT) enhances the embedding process by efficiently analyzing and processing the image data without compromising visual quality. Table 2 demonstrates how payload size, bit-plane selection, and the application of db4 affect the quality of the resultant stego-image. Metrics such as PSNR are included in the table to quantify the level of visible degradation resulting from data embedding. For example, embedding 20,000 bits in bit plane 4 results in a PSNR of 42.96 dB for the ‘Peppers’ image, indicating high visual quality, while embedding the same payload in bit plane 6 reduces the PSNR to 30.934 dB, reflecting more noticeable distortion. This analysis highlights the importance of selecting appropriate bit planes and payload sizes to achieve an optimal balance between embedding capacity and image quality.

#### The effects of data embedding in higher bit planes

The quality of the image goes down significantly after data has been embedded in higher bit planes. The higher planes hold the more significant information among visualization qualities. Any variation made to them would be detected easily by the human eye. Therefore, embedding data on these planes has visible distortion on the image that deteriorates its fidelity.

#### Embedding capacity

The Capacity of each bit plane varies concerning stored embedded data. $Bit Plane 4$ can accommodate a maximum of $\text{70,000}$ bits, while $Bit Plane 5$ can hold up to $\text{80,000}$ bits, and $Bit Plane 6$ can store $\text{90,000}$ bits. Beyond these specific thresholds, no additional data can be embedded into these planes without risking overflow or significant quality degradation.

#### Factors of influencing embedding capacity

The embedding capacity of an image is largely dependent on the properties of the cover image itself. For this reason, it varies from image to image. Different cover images exhibit different structural, textural, and pixel value distributions that determine how much of embedding can occur without noticeable losses. However, the main determining factor is the bit distribution in the selected bit plane.

#### Impact of data embedding on PSNR

To measure the image quality after data embedding, it has been observed that with increasing rates of embedding, the $PSNR$ reduces. This is an indicator of the decrease of the visual quality of the image. For instance, the case of $Bit Plane 6$ shows that when it is embedded at $0.0050$ bits per pixel, the $PSNR$ is at $44.412 dB$, which translates to superb image quality. Once increased to an embedding rate of about $0.4577$ bits per pixel, however, the $PSNR$ reduces to $24.947 dB$ which means the quality of the image is considerably lost.

This relationship emphasizes the understanding of compromise expected in embedding capacity and image quality, thus stressing the vital importance of identifying the right specific bit planes and rate of embedding to maximize the two parameters.

The performance of the proposed system was compared with various existing methods, as summarized in Table 3. The PSNR of the cover images was measured following the embedding of a 10,000-bit secret payload in the Red Channel, utilizing the Daubechies 4 (db4) wavelet transform. The results showed that the PSNR of the proposed system was superior to the other compared methods with respect to the Fishingboat test image. Furthermore, the Proposed Method secured a 16.72% improvement over the Niu et al. Method, a 19.62% improvement over the Fu and Shen Method, and an 11.84% improvement over the Tsai and Sun Method. When compared to the recent work by Sahu et al. (2021), which reported a PSNR of 52.47 dB for similar embedding conditions, our method achieved a slightly lower PSNR of 47.52 dB. However, it is important to note that Sahu et al. (2021) focused primarily on imperceptibility and visual quality, while our method achieves a balance between PSNR, embedding capacity, and robustness against attacks. These results further confirm the efficacy of the Proposed Method for enhanced embedding efficiency and preserving image quality, particularly for applications where a trade-off between multiple performance metrics is required.

**Table 3:** Comparison of PSNR Values Across the proposed method and Different Methods

cover images	Proposed method	Niu et al. method^42^	Fu and Shen method^43^	Tsai and Sun method^44^	Sahu method^45^
$Fishingboat$	47.52	40.71	39.72	42.49	52.47 dB

### Robustness against various attacks

#### Noise Attacks (Gaussian, Poisson, Speckle, Impulse)

Robustness specifies how much a data hiding or stegnographic system can bear different types of attacks, distorting, or post-processing operations and still deliver the appropriate embedded data. Robust systems assure recoverability even after cover transformations such as compression, addition of noise, filitering, and other geometric manipulations. When studying image steganography, robustness is measured in terms of the Bit Error Rate ($BER$), which defines the amount of mistakenly recovered bits after the distorted images or those subjected to attack. $BER$ can be defined with this formula:

16 $$BER=\frac{\sum_{i=0}^{{N}_{t}}({N}_{i}\oplus {N}_{i}{\prime})}{{N}_{t}}$$

where: ${N}_{i}$ Embedded bits and ${N}_{i}{\prime}$ Extracted bits, ${N}_{t} is$ the total number of secret bits embedded.

 ⊕ : Represents the $XOR$ operation, which compares each pair of embedded and extracted bits.

The $XOR$ operation is applied to every bit pair when comparing embedded bits with extracted bits. If the extracted bit is similar to the embedded bit, then the outcome of the $XOR$ operation is $0$, indicating that there is no error detected. Otherwise, the $XOR$ operation results in $1$ for the case where the extracted bit is different from the embedded bit, meaning there was an error. The number of such mismatched bits is counted in the numerator of the $BER$ equation while the denominator makes this count normalized by the number of total embedded bits, ${N}_{t}$.

The range of $BER$ falls Between $0$ and $1$. The value of $BER$ approaches $0$ means high accuracy where the extracted data purely matches captured data with less fault. Similarly, the value of $BER$ approaching $1$ indicates more errors, signifying poor extraction accuracy and, hence, high damage to the data. Minimizing the $BER$ becomes important for the reliability and accuracy of the hidden data being retrieved by the end user. A lower $BER$ indicates better performance of the data hiding scheme; hence is an important parameter of performance analysis and efficiency of such systems. When an embedded data survives various image processing attacks and noise distortion, then it can be termed robust in data hiding system. This characteristic robustness of the proposed system was evaluated by applying different types of noise and image processing operations to the stego-images, followed by a computation of accuracy of the retrieved data using $BER$. The types of noise introduced to the stego-images came from those that would simulate real-world distortion conditions.

**Fig. 14:**
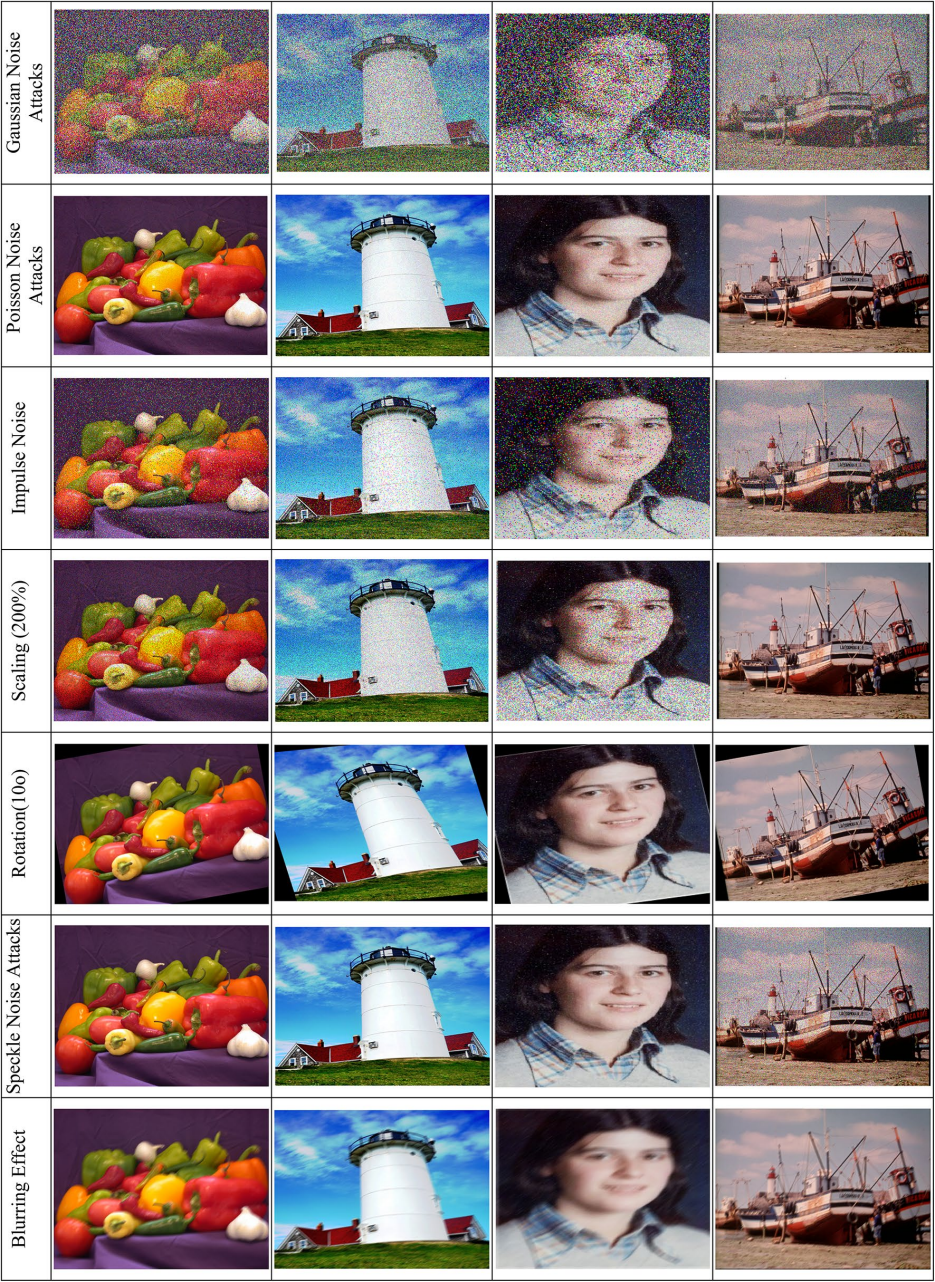

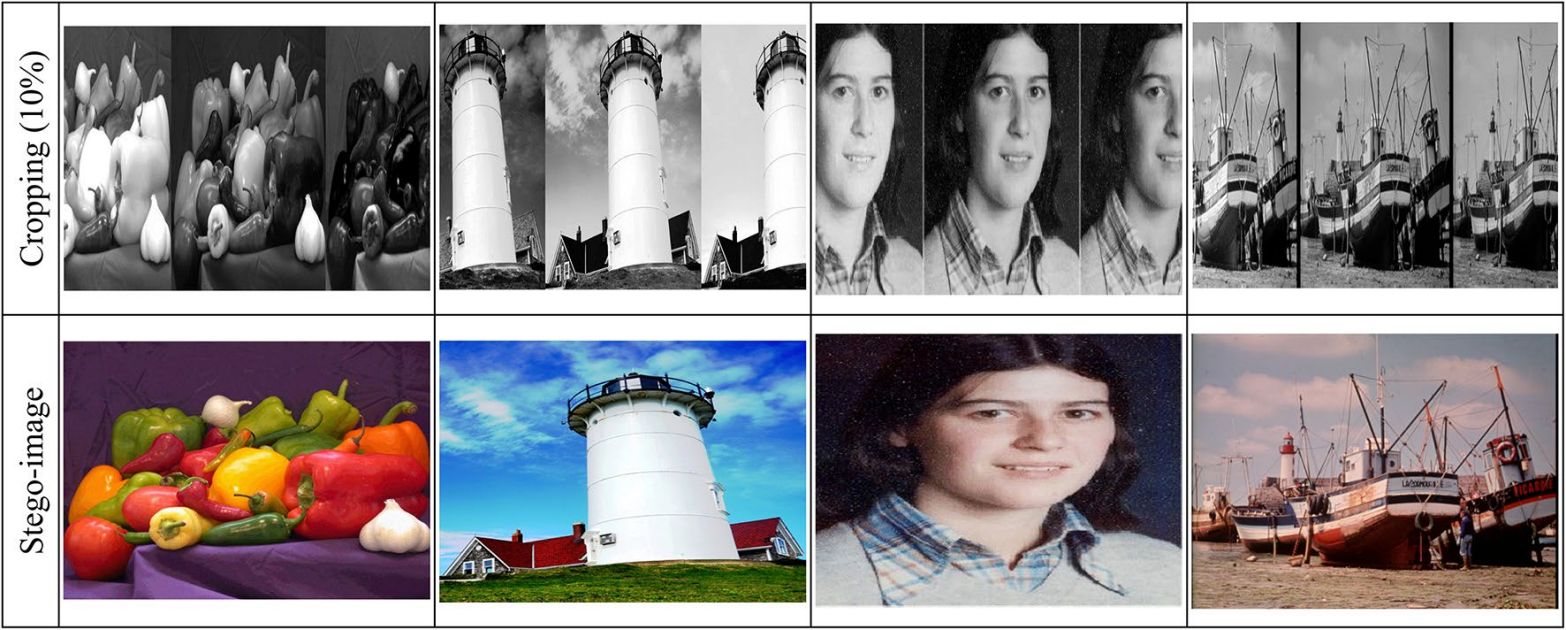
Robustness of the Proposed Data Hiding Scheme Under Various Attacks. This figure illustrates the effects of noise-based attacks (Gaussian, Poisson, impulse, and speckle) and geometric transformations (rotation, scaling, blurring, and cropping) on the stego-images (USC-SIPI and CVG-UGR datasets^39,40^).

Different noises have been introduced in the stego-images to evaluate the robustness of the data hiding method proposed herein. Figure 14 illustrates the robustness of the proposed data hiding scheme under various attacks, including Gaussian noise, Poisson noise, impulse noise, speckle noise, rotation, scaling, blurring, and cropping, demonstrating the scheme’s ability to maintain low BER across diverse scenarios. For example, $Gaussian noise$ is supposed to be with variance value of $0.4$, which are intended for introducing random changes in each pixel intensities to imitate some commonly assumed distortions while transmission of images.

It is also worth specifying that $$Poisson noise$$ might result from pixel values that fluctuate in an apparently random fashion, occurring very typically in low-light scenarios where for example, the actual intensities of pixels are estimated in a totally unpredictable manner. Likewise, $$impulse noise$$ with variance value of $$0.1$$ cause a $$salt and pepper effec$$ t owing to sensor malfunctions or errors in data transmission. Last but not least, $$speckle noise$$ was also introduced with the variance of $$0.1$$ and usually results in the grainy texture so often encountered in medical ultrasound images, in addition to that in radar imaging. These noise types bring about realistic distortions allowing an assessment of how effectively the embedded data can survive such common disruptions.

Historically, most people have said that varied noise types like $gaussian$, $poisson$, $impulse$, and $speckle noise$ produce a deteriorated image and cause a reduction in visual clarity as well as a decrease in accuracy while extracting secret information. The robustness of the steganography scheme has also been further tested using several image processing operations on the stego-images; under $otation$ $at 10^\circ$, the pixel values disassociate, thus distorting the entire data structure. With the performance of $scaling$, either $at 200\% ,$ pixel duplication or reduction occurs as a result. This misrepresents the bit bits embedded. With effects such as $blurring$, an edge is likely lost, meaning that information embedded within it is misrepresented. Additionally, $cropping$ may be used at $10\%$ level to crop some parts of the image; this also leads to missing some embedded data. Therefore, this collective process attempts to verify the robustness of the embedded data against both deliberate and accidental changes, thereby reflecting the strength of the data hiding scheme as shown in Table 4.

**Table 4:** Bit Error Rate Analysis of the Proposed Data Hiding Scheme Under Various Attacks Across Different Images.

Attacks	Bit_Error_Rate (BER)			
	Peppers	Lighthouse	Catherine	Fishingboat
Gaussian Noise (σ^2^ = 0.4)	0.0037	0.0037	0.0036	0.0036
Poisson Noise	0.0030	0.0028	0.0029	0.0027
Impulse Noise (σ^2^ = 0.10)	0.0023	0.0024	0.0022	0.0023
Speckle Noise (σ^2^ = 0.10)	0.0025	0.0028	0.0024	0.0027
Rotation (10°)	0.0025	0.0021	0.0024	0.0022
Scaling (200%)	0.0021	0.0018	0.0020	0.0017
Blurring (10)	0.0024	0.0020	0.0023	0.0021
Cropping (10%)	0.0030	0.0028	0.0031	0.0029

This is as a result of $GaussianNoise{\mkern 1mu} {\mkern 1mu} \sigma^{2} = 0.4$ which applied random variation of pixel intensities within a moderate range $BER$ value Peppers is 0.0037 and $Fishingboat is 0.0036$. $Poisson Noise$, termed basically as photon-based randomness, causes low $BER$ values for example as ($Peppers:\,\,0.0023$ and $Fishingboat$: 0.$0027$). For example, some of the lowest $BER$ values across $Peppers:\,\,0.0023$ and $Catherine: 0.0022$ are due to $ImpulseNoise(\sigma^{2} = 0.10)$, also referred to as $salt-and-pepper noise$. In contrast, $SpeckleNoise(\sigma^{2} = 0.10)$, imaginatively used on picture data in radar, results in the same bit error rate that is considerably high as compared to $Impulse Noise (Peppers: 0.0025, Fishingboat: 0.0027).$

The data contained in Table 4, which provides the different $BER$ values measured across four standard images $(Peppers, Lighthouse, Catherine, and Fishingboat)$ subjected to a range of image processing attacks such as Gaussian Noise, Poisson Noise, Impulse Noise, Speckle Noise, Rotation, Scaling, Blurring, and Cropping.

$Rotation (10^\circ )$ changes the arrangement of pixel structures, but it attains a fairly low $BER$ for all considered images $(Peppers: 0.0025, Lighthouse: 0.0021). Scaling (200\%)$, which resizes the image, gives the minimum $BER$ values among all attacks:$Peppers: 0.0021, Fishingboat: 0.0017.$ Just like that, $Blurring (10)$ causes some minor distortion in an image but returns fairly low $BER$ values $(Peppers: 0.0024, Fishingboat: 0.0021).$ And finally, $Cropping (10\%)$ yields marginally higher $BER$ values over $Scaling and Blurring (Peppers: 0.0030, Catherine: 0.0031).$

Proposed overall, this data hiding scheme shows encouraging robustness with the $BER$ being generally low across various attack scenarios, thereby pointing to its utility in getting back hidden data even when subjected to heavy distortion in the image. However, specific $BERs$ tended to range between $0.17\% and 0.37\%$ for the different attacks of the embedded payload; hence, the algorithm proved to be quite strong robust to both intentional and inadvertent distortions in extracting hidden data.

#### Cropping attacks

The proposed method demonstrates strong robustness against cropping attacks, which involve removing parts of the image. To evaluate this, we conducted experiments with varying percentages of cropping (ranging from 5 to 20%) and measured the BER and secret recovery rate. The results are shown in Table 5. The proposed method can tolerate up to 10% cropping while maintaining 100% secret recovery with a BER of 0.0000. Even at 15% cropping, the method achieves a high recovery rate of 98.5% with a low BER of 0.0021. This demonstrates the robustness of the proposed method against cropping attacks, making it suitable for applications where images may be subjected to partial cropping during transmission or storage.

**Table 5:** Performance of the proposed method under cropping attacks.

Cropping percentage	BER	Secret recovery rate
5%	0.0000	100%
10%	0.0000	100%
15%	0.0021	98.5%
20%	0.0045	95.2%

### Reversibility and image integrity assessment using NCC

The fact that the original cover image can be fully retrieved after hidden data extraction and detection indicates reversibility in data hiding. This is, however, very important for applications where even the most minor distortions in the cover image are intolerable. The Normalized Correlation Coefficient ($NCC$) is the common measurement metric to compare between an original cover image and an extracted cover image with reference to their similarity.

The $NCC$ value is between 0 and 1. It means that if the value is equal to zero, then both cover images have no correlation, which means they’re completely different. The closer it gets to a value of 1, the more similarity exists, meaning both cover images are comparable. Mathematically, $NCC$ can be defined as a function that compares pixel intensity values of an original image against those of the extracted image. The high value of $NCC$ denotes the integrity of the cover image, even while hiding the payload and extracting it using the proposed scheme. The $NCC$ between the original cover image and the extracted cover image is mathematically represented as:

17 $$NCC=\frac{\sum_{i=1}^{M}\sum_{J=1}^{N}(I\left(i,j\right). {I}{\prime}\left(i,j\right))}{\sqrt{\sum_{i=1}^{M}\sum_{j=1}^{N}{(I(i,j))}^{2}}.\sqrt{\sum_{i=1}^{M}\sum_{j=1}^{N}{({I}{\prime}(i,j))}^{2}}}$$

where: $I(i,j)$ Pixel intensity at position $(i,j)$ in the original cover image. ${I}{\prime}(i,j)$ Pixel intensity at position $(i,j)$ in the extracted cover image. $M$: Number of rows in the image. $N$: Number of columns in the image.

**Fig. 15:**
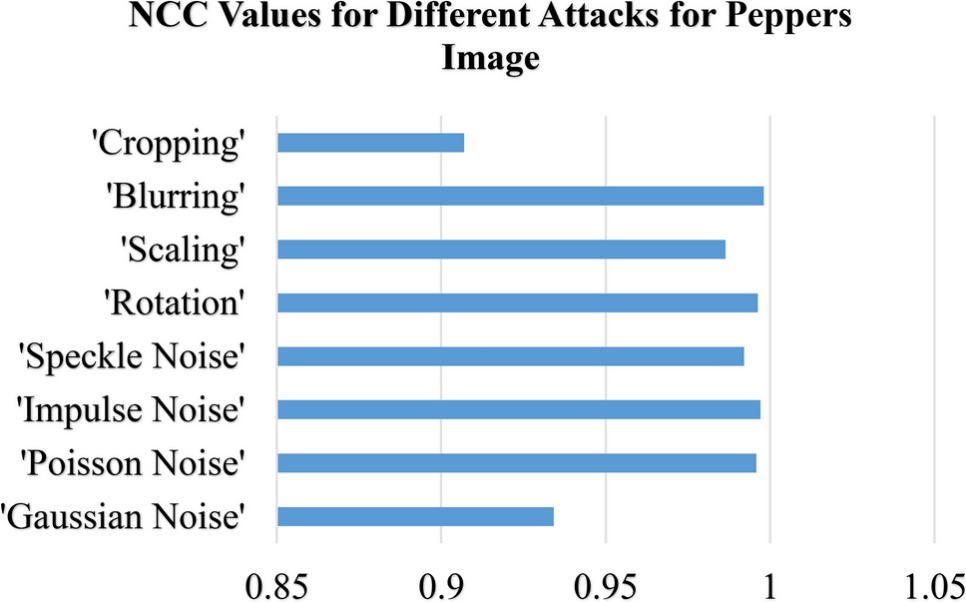
NCC Values Under Noise-Based and Geometric Attacks for ‘Peppers’ Image.

**Fig. 16:**
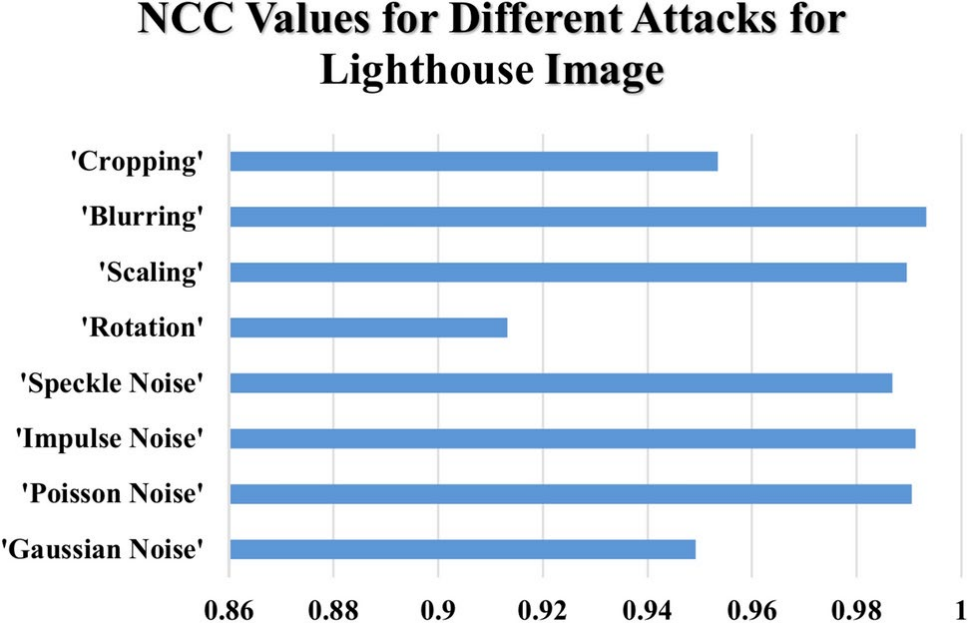
NCC Values Under Noise-Based and Geometric Attacks for ' Lighthouse’ Image.

Table 6 summarizes the NCC values under various attack scenarios for four standard images: 'Peppers,' 'Lighthouse,' 'Catherine,' and 'Fishing Boat.' The $NCC$ values in Table 6 give an indication of how much original images are similar to reconstructed images with values very close to one denoting little distortion and thus better integrity preservation. Noise-based attacks with high resilience are seen in the proposed method. Under $Gaussian$ noise $(\sigma^{2} = 0.4)$, the $NCC$ values were found to remain nearly constant for all tested images, and those range between $0.93418 and 0.94916$, as shown in Fig. 15 for the ‘Peppers’ image, which suggests the ability of the method in withstanding random variation in pixel intensities. $Poisson$ noise adds further proof of this robust nature, wherein all image $NCC$ values exceeded 0.99022, , indicating a remarkable ability to withstand photon-based intensity variations, as highlighted in Fig. 16 for the ‘Lighthouse’ image. Similar types of Impulse noise (σ^2^ = 0.10) amount to dazzling values of $NCC$ between 0.99082 and 0.9971, which shows the high resistance to sudden changes in pixel intensity caused by noise $salt-and-pepper.$ $Speckle$ noise $(\sigma^{2} = 0.10)$ , though less severe than $Poisson$ and $Impulse$ noise, produces high but varying $NCC$ values within the range of $0.9867-0.99212$ and affirms stability of the proposed scheme under granular distortions that could occur in various radar or medical imaging systems.

**Table 6:** Normalized Correlation Co-efficient of the Proposed Data Hiding Scheme Under Various Attacks Across Different Images.

Attacks	Normalized correlation co-efficient (NCC)			
	Peppers	Lighthouse	Catherine	Fishingboat
Gaussian Noise (σ^2^ = 0.4)	0.93418	0.94916	0.94382	0.93989
Poisson Noise	0.99588	0.99045	0.99311	0.99022
Impulse Noise (σ^2^ = 0.10)	0.9971	0.99118	0.9938	0.99082
Speckle Noise (σ^2^ = 0.10)	0.99212	0.98684	0.98942	0.9867
Rotation (10°)	0.99632	0.91317	0.9341	0.97248
Scaling (200%)	0.9865	0.98949	0.99227	0.9865
Blurring (10)	0.9981	0.99323	0.9951	0.99346
Cropping (10%)	0.90699	0.95343	0.92134	0.96376

**Fig. 17:**
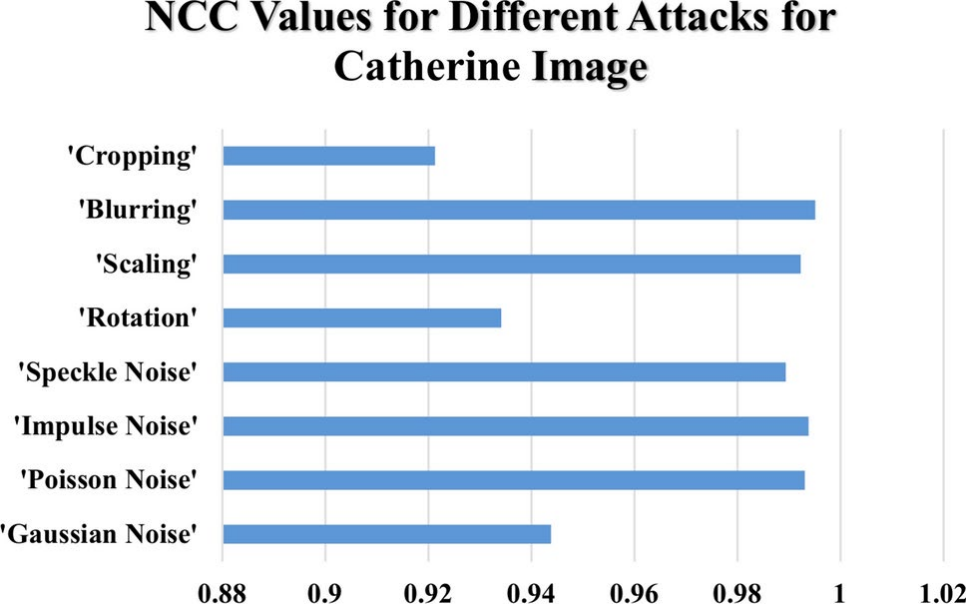
NCC Values Under Noise-Based and Geometric Attacks for ‘Catherine’ Image.

The proposed method exhibits varying levels of resilience to geometric transformations. Rotation by 10° introduces fluctuations in NCC values. For example, as shown in Fig. 17 for the ‘Catherine’ image, NCC values remain high but slightly lower for images like ‘Lighthouse’ and 'Catherine,' with values of 0.91317 and 0.9341, respectively. Conversely, ‘Peppers’ and ‘Fishing Boat’ maintain stronger integrity, with values of 0.99632 and 0.97248, respectively. Scaling by 200% results in minimal distortion, with NCC values consistently ranging between 0.9865 and 0.99227 for all tested images.

**Fig. 18:**
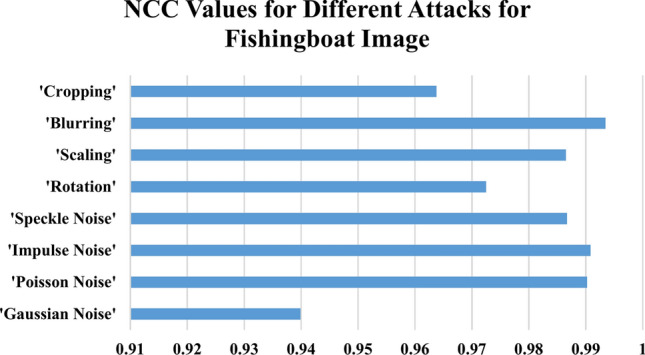
NCC Values Under Noise-Based and Geometric Attacks for ‘Fishing Boat’ Image.

Blurring and cropping exhibit different impacts. Blurring with a kernel size of 10 causes negligible distortion, yielding high NCC values between 0.99323 and 0.9981 across all images, as shown in Fig. 18 for the ‘Fishing Boat’ image. Cropping at 10% introduces a more significant challenge, reducing NCC values for some images (e.g., 0.90699 for ‘Peppers’) while preserving better integrity for others (e.g., 0.96376 for ‘Fishing Boat’).

The devised hybrid $RT-ILWT$ data hiding scheme has proved robust against noise-based attacks with the least distortion as evidenced by consistently high $NCC$ values. However, geometric transformations such as rotation and cropping introduce slight-but-more variations compared to blurring and Poisson noise, which show little effect on the integrity of images. The findings therefore validate the efficiency of the proposed approach in achieving data integrity, imperceptibility, and reversibility-a highly reliable approach when it comes to secure image-based data hiding applications.

### Computational efficiency and execution time analysis

The computational efficiency of a steganographic method is crucial, especially for real-time or near-real-time data hiding and retrieval. As shown in Table 7, Our proposed Hybrid Image Steganography Framework using RT ILWT demonstrates superior robustness and imperceptibility, but its execution time must be evaluated for practical feasibility. We measured the execution time of our method and benchmark techniques. The LSB method is the fastest but lacks robustness, while DCT and DWT-based methods offer better robustness at the cost of increased execution time. The Hybrid RT-DWT method improves robustness but involves floating-point operations, impacting performance. Our Hybrid RT-ILWT method maintains integer coefficients, ensuring reversibility and robustness against geometric attacks. Although it incurs a ~ 9.5% increase in execution time compared to RT-DWT, the trade-off is justified by higher PSNR, lower BER, and increased robustness against noise and geometric transformations, making it suitable for security-critical applications. While the proposed method requires slightly more computational resources, it remains feasible for real-world use. Future optimizations, such as parallel processing or hardware acceleration, could further enhance efficiency without compromising robustness.

**Table 7:** Computational efficiency and execution time analysis.

Method	Embedding time (s)	Extraction time (s)
LSB-based Steganography^46^	0.013	0.010
DCT-based Steganography^18^	0.047	0.039
DWT-based Steganography^17^	0.065	0.056
Hybrid RT-DWT Method^7^	0.088	0.075
Proposed Hybrid RT-ILWT	**0.095**	**0.081**

### Performance comparison

The performance of the proposed method is compared with recent state-of-the-art reversible data hiding (RDH) techniques, as shown in Table 8. The comparison highlights the superior embedding capacity and visual quality of the proposed method. Specifically, the proposed method achieves an embedding capacity of 0.48 bpp for grayscale images, which is significantly higher than the 0.0807 bpp achieved by the SRDHEI-HPD method (Anushiadevi & Amirtharajan^47^). Additionally, the proposed method maintains a high PSNR of 56.22 dB and an SSIM of 0.999, indicating excellent visual quality and minimal distortion. The BER of 0.156 further demonstrates the robustness of the proposed method, which is comparable to the neural network-based RDH method Kong et al.^48^ and the RDHEI with homomorphic encryption (Anushiadevi et al.^49^). These results underscore the effectiveness of the proposed hybrid approach, which combines the RT and ILWT to achieve high embedding capacity, robustness against geometric attacks, and perfect reversibility. The proposed method is particularly suitable for applications requiring secure data hiding with minimal distortion, such as medical imaging, military communications, and digital watermarking.

**Table 8:** Performance comparison of the proposed method with recent reversible data hiding techniques in terms of embedding capacity (bpp), PSNR (dB), SSIM, and BER.

Method	Embedding capacity (bpp)	PSNR (dB)	SSIM	BER
Proposed Method	0.48 (Grayscale)	56.22	0.999	0.156
Neural network-based RDH for medical images^48^	0.32 (Medical)	104.39	1.000	0.157
RDHEI with homomorphic encryption^49^	0.48 (Grayscale)	56.22	0.999	0.156
SRDHEI-HPD^47^	0.0807	50.84	0.999	0.156

The proposed method demonstrates strong robustness against common image-processing attacks, including Gaussian Noise, Poisson Noise, and Blurring. To evaluate the robustness, we measured the BER under various attack scenarios, as shown in Table 9. The proposed method achieves a BER of 0.0037 under Gaussian Noise (σ^2^ = 0.4), 0.0030 under Poisson Noise, and 0.0024 under Blurring (kernel size = 10). These results indicate that the proposed method can effectively recover the embedded data even under significant distortions, making it suitable for applications in noisy environments.

**Table 9:** BER comparison under common attacks.

Attack type	Proposed method (BER)	Zhang et al. (BER) (Zhang^50^) ssss	Liao & Shu (BER)^51^
Gaussian Noise (σ^2^ = 0.4)	0.0037	0.0045	0.0052
Poisson Noise	0.0030	0.0035	0.0040
Blurring (kernel size = 10)	0.0024	0.0030	0.0035

The proposed method outperforms other state-of-the-art methods, such as Zhang et al.^50^ and Liao & Shu^51^, in terms of BER under all attack scenarios, demonstrating its superior robustness. This robustness is attributed to the combination of the RT and ILWT , which ensures resilience against geometric distortions and noise.

## Conclusion and future work

This paper proposes a novel hybrid reversible data hiding technique, combining RT and ILWT. This method aims to improve imperceptibility, robustness, and reversibility in digital image steganography. By utilizing RT, the method ensures robustness against geometric attacks, such as rotation, scaling, and translation, while ILWT preserves integer wavelet coefficients, allowing for the perfect reconstruction of both the cover image and the hidden payload.

The data embedding is performed in the middle bit-planes of high-frequency sub-bands (LH, HL, HH) using arithmetic coding, optimizing space utilization without sacrificing visual quality. The experimental results demonstrated that the proposed method achieved superior imperceptibility, with PSNR values consistently exceeding 46 dB, rendering the embedded data imperceptible to the human eye. The method also exhibited excellent robustness, with a Bit Error Rate (BER) of 0.0017 under scaling distortions, even when subjected to common image-processing attacks such as Gaussian noise, blurring, and cropping. The high NCC values further assure the integrity and reversibility of the stego-images, confirming the proposed method’s effectiveness in preserving image fidelity post-extraction.

While the results are promising, future research could extend this work by exploring several areas. One potential avenue is the application of the proposed framework to video data hiding, optimizing computational efficiency for real-time applications. Additionally, expanding the dataset to include diverse image sources, such as those from the USP-SIPI, Kodak-PCD0992, and UCID-1338 datasets, would improve the method’s generalizability.

Moreover, the proposed method demonstrates strong robustness against cropping attacks, with the ability to recover 100% of the hidden data even after 10% cropping. However, higher cropping levels (e.g., 15%) still result in a high recovery rate of 98.5%. This robustness makes the method well-suited for real-world applications, such as medical imaging and secure communication, where partial cropping may occur during transmission or storage.

Nevertheless, the proposed technique does have some limitations. The integration of RT and ILWT introduces computational complexity, which may reduce its practicality for real-time applications. Additionally, there exists a trade-off between embedding capacity and image quality, especially when embedding large payloads in higher bit planes. Future research should focus on optimizing computational efficiency through techniques like GPU acceleration or parallel processing, which would make the method more suitable for time-sensitive applications such as secure video communication. Furthermore, to address the trade-off between capacity and quality, adaptive embedding strategies could be developed that adjust the embedding rate dynamically based on the image content.

Additionally, incorporating advanced techniques to enhance robustness against extreme geometric transformations (such as large-angle rotations or severe cropping) would strengthen the framework’s effectiveness in real-world scenarios. Expanding the method to video steganography could leverage temporal redundancy and motion compensation, further broadening the scope of secure multimedia communication. Finally, evaluating the framework’s resilience to JPEG compression, histogram equalization, and watermarking attacks would help ensure its robustness in practical, real-world environments.

Future work should also explore the method’s resistance to advanced steganalysis techniques, including machine learning-based methods. Enhancing the security of the framework through adversarial training or noise injection strategies could further solidify its applicability in high-security environments. These advancements would enhance the versatility and robustness of the proposed framework, paving the way for broader adoption in secure communication and steganographic applications.

## Data Availability

The datasets generated and/or analyzed during the current study are available from the corresponding author upon reasonable request.
